# A systematic study of hexavalent chromium adsorption and removal from aqueous environments using chemically functionalized amorphous and mesoporous silica nanoparticles

**DOI:** 10.1038/s41598-020-61505-1

**Published:** 2020-03-27

**Authors:** Eun-Hye Jang, Seung Pil Pack, Il Kim, Sungwook Chung

**Affiliations:** 1 School of Chemical and Biomolecular Engineering, Pusan National University, 2 Busandaehak-ro 63beon-gil, Geumjeong-gu, Busan 46241 South Korea; 2 Department of Biotechnology and Bioinformatics, Korea University, 2511 Sejong-Ro, Sejong, 30019 South Korea; 3 Department of Polymer Science and Engineering, Pusan National University, 2 Busandaehak-ro 63beon-gil, Geumjeong-gu, Busan 46241 South Korea

**Keywords:** Nanoparticles, Characterization and analytical techniques, Pollution remediation, Nanoparticle synthesis, Synthesis and processing, Nanoparticles

## Abstract

We report on the synthesis and characterization of highly monodisperse amorphous silica nanoparticles (ASNs) and mesoporous silica nanoparticles (MSNs) with particle sizes of 15–60 nm. We demonstrate adsorption of Cr(VI) ions on amino-functionalized ASNs (NH_2_–ASNs) and MSNs (NH_2_–MSNs) and their removal from aqueous environments and show the specific surface area (SSA) of NH_2_–MSNs is four times as larger as that of NH_2_–ASNs and that more than 70% of the total SSA of NH_2_–MSNs is due to the presence of nanopores. Analyses of Cr(VI) adsorption kinetics on NH_2_–ASNs and NH_2_–MSNs exhibited relatively rapid adsorption behavior following pseudo-second order kinetics as determined by nonlinear fitting. NH_2_–ASNs and NH_2_–MSNs exhibited significantly higher Cr(VI) adsorption capacities of 34.0 and 42.2 mg·g^−1^ and removal efficiencies of 61.9 and 76.8% than those of unfunctionalized ASNs and MSNs, respectively. The Langmuir model resulted in best fits to the adsorption isotherms of NH_2_–ASNs and NH_2_–MSNs. The adsorption of Cr(VI) on NH_2_–ASNs and NH_2_–MSNs was an endothermic and spontaneous process according to the thermodynamic analyses of temperature-dependent adsorption isotherms. The removal efficiencies of NH_2_–ASNs and NH_2_–MSNs exhibited a moderate reduction of less than 25% of the maximum values after five regeneration cycles. Furthermore, NH_2_–MSNs were also found to reduce adsorbed Cr(VI) into less harmful Cr(III).

## Introduction

Chromium is used for many purposes in modern industries. For example, it is used to harden steel and make stainless steel, which are essential raw materials in the construction, heavy machinery, automotive, transportation, energy, and medical industries. Chromium is also used extensively in chrome electroplating processes, in paint pigments, and to produce dyes, leather, plastics, and photographs. These industrial activities generate substantial amounts of hazardous wastes containing relatively high concentrations of chromium, which is recognized as one of the most toxic inorganic pollutants due to its mutagenic and carcinogenic effects on biological species^1,2^.

In aqueous environments, chromium generally exists in the trivalent (Cr(III)) or hexavalent (Cr(VI)) oxidation states. Ionic species containing Cr(VI) such as CrO_4_^2−^ or HCrO_4_^−^ are highly soluble and mobile in nature than Cr(III), and thus, Cr(VI) containing species pass through cell membranes readily, whereas Cr(III) species are poorly cell-permeable^3^. In fact, it has been estimated Cr(VI) is almost ~100 times more toxic than Cr(III) in aqueous environments^4,5^. Sequestration strategies targeting the adsorption and reduction of Cr(VI) to less harmful Cr(III) attracted considerable research attention because of their obvious advantages over strategies aimed at the separation of Cr(VI)^6^.

Methods such as adsorption^7–9^, ion-exchange^10,11^, membrane separation^12,13^, coagulation^14^, chemical precipitation^15,16^, extraction^17^, dialysis^18^, and electrochemical separation^19^ have been shown to be capable of removing heavy metal ions, including hexavalent chromium from wastewater. Of these different methods, adsorption is probably the most effective, economically feasible, environmentally sustainable, and technologically promising processes^20^. Recently, a variety of adsorbents such as activated carbons^7,21^, iron-based metal oxides^22^, metal-organic frameworks^23^, polymeric and biomass-based materials^9,24,25^ have been used for Cr(VI) removal. In particular, polymeric materials have been shown to be highly efficient at adsorbing Cr(VI) and their highly branched structures can be readily functionalized to selectively adsorb and remove different heavy metal ions^26,27^.

Silica and silica-based composite materials are an essential class of adsorbents that have been widely employed due to their unique and adjustable physicochemical characteristics, which include large surface areas and excellent chemical, thermal, and mechanical stabilities^27^. Stöber silica is often used as an adsorbent and generally described as a nonporous, monodisperse, and amorphous silica composed of spherical particles^28^. A variety of methods such as sol-gel, microemulsion, hydrothermal, and flame-based methods have been devised to synthesize silica particles with diameters in the colloidal range^29^. Over the past years, a collection of mesoporous silica, such as SBA-1^30^, SBA-15^31^, MCM-41^32^, and MCM-48^33^ have been synthesized and also used as adsorbents that have highly ordered nanoscale pores with a wide range of pore geometries including hexagonal and cubic arrangements with relatively narrow size distributions. Mesoporous silica with uniform and tailorable pore dimensions exhibits unique material properties such as high specific surface areas and excellent thermal and mechanical stabilities, and hence, has been employed in potential applications^34^. These include their use in heavy metal removal from wastewater^6^, indoor air purification^35^, CO_2_ capture^36^, pervaporation membranes for the separation of water from ethanol^37^.

Both Stöber and mesoporous silica materials have been chemically functionalized to enhance Cr(VI) adsorption^38,39^. For example, nitrogen-containing functional groups like aliphatic and aromatic amines have demonstrated to be extremely effective at enhancing Cr(VI) adsorption^27^. It has been suggested that the adsorption behavior of Cr(VI) on functionalized silica surfaces depends on parameters such as the number densities and the binding strengths of the donor groups grafted onto adsorbent surfaces^40,41^. However, little is known of the processes involved, such as of the effect of specific functional groups, or of relationships between adsorption efficiencies and adsorbent sizes, shapes, morphologies, pore densities, and specific surface areas and many aspects of the processes have yet to be explored systematically.

Herein, we present the results of work aimed at preparing two types of bare and two types of NH_2_-functionalized silica nanoparticles of similar sizes, that is, amorphous silica nanoparticles (ASNs) with an amorphous solid core and mesoporous silica nanoparticles (MSNs) with a nanoporous core. Also, we investigated their Cr(VI) adsorption and removal performances using aqueous batch systems. N_2_ sorption measurements showed the specific surface areas (SSAs) of unfunctionalized MSNs were twice as large as those of ASNs, and that the SSAs of 3-aminopropyl triethoxysilane (APTES) functionalized MSNs (NH_2_–MSNs) were four times as large as those of APTES functionalized ASNs (NH_2_–ASNs). In addition, we confirmed that> 70% of the total SSAs of bare and NH_2_-functionalized MSNs were ascribable to nanopores using a simplified geometric scaling approach. We examined the effect of temperature and pH on adsorption and removal of Cr(VI) using bare and NH_2_-functionalized ASNs and MSNs. We discovered that unfunctionalized ASNs and MSNs had considerably lower adsorption capacities that were less than ~4% of those of NH_2_–ASNs and NH_2_–MSNs. Furthermore, we confirmed that the optimal pH for reaching maximum Cr(VI) uptake and removal efficiency were 2.0. Systematic analyses of Cr(VI) adsorption kinetics using non-linear fitting of a pseudo-second order kinetic model at pH 2.0 and 25 °C revealed rapid adsorption of Cr(VI) on NH_2_–ASNs and NH_2_–MSNs whereby ~90% of Cr(VI) was adsorbed within one minute. In-depth analyses of the equilibrium adsorption isotherms of NH_2_–ASNs and NH_2_–MSNs revealed that Langmuir model resulted in the best fit to the experimental data. NH_2_–ASNs and NH_2_–MSNs exhibited significantly higher Cr(VI) adsorption capacities (*q*_*e*_) of 34.0 and 42.2 mg·g^−1^ and removal efficiencies (*R*) of 61.9 and 76.8% than those of unfunctionalized ASNs and MSNs (0.4 and 1.3 mg·g^−1^ and 0.7 and 2.4%), respectively. Furthermore, we found that NH_2_-functionalized ASNs and MSNs could also reduce adsorbed Cr(VI) to Cr(III) and that the amount of Cr(VI) reduction adsorbed on NH_2_–MSNs was three times as large as those on NH_2_–ASNs.

## Materials and Methods

### Materials

Tetraethyl orthosilicate (TEOS, ≥ 99%, Aldrich), cetyltrimethyl ammonium chloride (CTAC, 95%, Wako), triethanolamine (TEA, ≥ 99%, Aldrich), 3-aminopropyl triethoxysilane (APTES, 99%, Aldrich), potassium dichromate (K_2_Cr_2_O_7_, ≥99.5%, KANTO), and 1,5-diphenylcarbazide (DPC, special GR grade, SAMCHUN) were purchased and used as supplied. All other chemicals including the solvents used such as toluene, acetone, absolute ethanol, 1 N sulfuric acid, hydrochloric acid solution (10 wt%), and ammonium hydroxide (NH_4_OH) solution (25 wt%) were of analytical ACS regent grade and once purchased, they were used as supplied.

### Preparation of monodisperse ASNs

Stöber *et al*. originally reported the synthesis of ASNs via a base-catalyzed hydrolysis and condensation of TEOS^42^. This method and its modifications have been widely used to synthesize monodisperse ASNs^43,44^. We synthesized ASNs using a modified version of the original method. Typically, 4.43 mL TEOS was added dropwise to a pre-mixed solution of 1.35 mL doubly distilled deionized water (18.2 MΩ·cm), 200 mL absolute ethanol, and 2.25 mL 25 wt% NH_4_OH solution with stirring at 400 rpm. The resulting suspension was immediately stirred for 24 h at 25 °C and then dialyzed against 5 L of doubly distilled deionized water using a dialysis cellulose membrane tubing (pore size of 2.5–5 nm, Sigma Aldrich) to remove ethanol and ammonia from the suspension. The dialysate was refreshed 4 times at 2 h intervals. Finally, ASNs were precipitated by rotaevaporation for 30 min, filtered through a 0.2 μm nylon membrane, washed with an excess of absolute ethanol, and dried in a vacuum oven at 25 °C.

### Preparation of monodisperse MSNs

Bein *et al*. reported the synthesis of MSNs using TEA as a complexing agent for silicic precursors and CTAC as a templating agent for inner pores within ASNs^45^. We synthesized highly monodisperse MSNs with sizes that closely matched those of as-prepared ASNs prepared in a similar manner^46^. Briefly, 0.5 g CTAC and 0.06 g TEA were dissolved in 20 mL doubly distilled deionized water at 80 °C with stirring at 700 rpm for 1 h. 1.5 mL of TEOS was then added dropwise at a rate of 1 mL/min. The resulting suspension was then immediately stirred for 1 h at 80 °C and as-synthesized MSNs were precipitated by centrifugation at 12,000 rpm for 15 min, washed with an excess of absolute ethanol to remove residual reactants, and dried in a vacuum oven at 25 °C. Finally, MSNs were calcined at temperatures up to 550 °C for 4 h using a heating rate of 10 °C·min^−1^ to remove CTAC from inner pores.

### Surface functionalization of ASNs and MSNs with APTES

ASNs and MSNs were functionalized with APTES using a modification of a previously described method^6,47^. Approximately ~1 g of as-synthesized ASNs or calcined MSNs were reacted with 1 mL of APTES separately in 100 mL of toluene at 80 °C with vigorous stirring overnight. Products were removed by filtration, washed with an excess of absolute ethanol, and doubly distilled deionized water to remove most of the unreacted APTES, and dried in air at 25 °C. Because this washing step was not enough to remove all the unreacted and physisorbed APTES, products were re-immersed in doubly distilled deionized water (at sample/water ratio 1 g/L) with stirring for at least 24 h at 30 °C^48^. Finally, products were filtered, washed with an excess of absolute ethanol and doubly distilled deionized water, and dried in a vacuum oven at 25 °C.

### Scanning electron microscopy (SEM), transmission electron microscopy (TEM), X-ray powder diffraction (XRD)

Sizes and morphologies of ASNs and MSNs were investigated by field emission scanning electron microscopy (FESEM) using a Zeiss Supra 40 FESEM unit at an accelerating voltage of 5 to 10 kV and by transmission electron microscopy (TEM) using a Hitachi H-7600 TEM operating at an accelerating voltage of 80 kV. High-resolution TEM (HRTEM) and scanning TEM (STEM) analyses were performed on an FEI TALOS F200X TEM operating at an accelerating voltage of 200 kV. The chemical compositions of ASNs and MSNs were analyzed simultaneously using an energy dispersive X-ray spectroscopy (EDS) detector attached to Zeiss Supra 40 FESEM. X-ray powder diffraction (XRD) patterns were produced using a Philips X’Pert-MPD Diffractometer equipped with a monochromatic X-ray of Cu K_α1_ radiation (λ = 0.15405 nm) at 1.6 kW (40 kV, 30 mA).

### Fourier transform infrared spectroscopy (FT-IR) and thermogravimetric analysis/differential thermal analysis (TGA/DTA)

ASNs, MSNs, NH_2_–ASNs, and NH_2_–MSNs were subjected to Fourier transformation infrared spectroscopy (FT-IR) using a Spectrum GX FT-IR spectrometer and KBr pellet and attenuated total reflection (ATR) techniques. Thermal analyses of NH_2_–ASNs and NH_2_–MSNs were carried out using a TA SDT Q600 thermal gravimetric analysis/differential thermal analysis (TGA/DTA) instrument. Typically, ~5 mg of sample was heated in an Al_2_O_3_ crucible at a heating rate of 5 °C/min under N_2_ flow of 100 mL/min for up to 750 °C.

### X-ray photoelectron spectroscopy (XPS)

XPS data of ASNs, MSNs, NH_2_–ASNs, and NH_2_–MSNs were acquired using an ESCALAB 250 spectrometer equipped with a monochromatic X-ray source of Al anode K_α_ radiation (1486.6 eV) used as an excitation source. Binding energy was calibrated with respect to the C 1 s line of carbon at 284.6 eV before actual measurements.

### Nitrogen (N_2_) physisorption experiments for Brunauer-Emmett-Teller (BET) and Barrett-Joyner-Halenda (BJH) analysis

N_2_ adsorption-desorption measurements were performed on a Quantachrome Autosorb-iQ surface area and pore size analyzer. Samples of ASNs, MSNs, NH_2_–ASNs, and NH_2_–MSNs were degassed at 300 °C for ~3 hours before actual measurements. BET analysis was used to calculate specific surface areas. Pore size distributions were calculated by BJH analysis of the isotherms’ desorption branches.

### Reagents and standard preparations for UV-Visible spectrophotometric analysis (a.k.a. the DPC method)

A standard stock solution of Cr(VI) was prepared by dissolving 2.829 g of dried K_2_Cr_2_O_7_ in doubly distilled deionized water (18.2 MΩ cm) and then diluting further to 1000 mL and a 100 mg/L solution of Cr(VI) was prepared by diluting the standard stock solution. Separately, a 1,5-diphenylcarbazide (DPC) stock solution was prepared by dissolving 50 mg of DPC in 25 mL of acetone containing 250 μL of 1 N sulfuric acid^49,50^. Working standard Cr(VI) concentrations of 1, 10, 20, 50, and 100 mg/L were prepared from the standard stock solution and their pH values were adjusted to ~2.0 with a hydrochloric acid solution (10 wt%). Then, 3 mL aliquots from working standard solutions were mixed with 100 μL DPC stock solution to form Cr(VI)-DPC complexes, which were quantified by spectrophotometry (λ_max_ = 540 nm) at equilibrium. The standard absorbance *vs*. concentration calibration plot was determined by linear regression to have a correlation coefficient (*R*^2^) of 0.9969 and used to determine Cr(VI) concentrations together with the results from inductive coupled plasma optical emission spectroscopy (ICP OES) before batch and kinetic batch experiments.

### Batch and kinetic batch experiments on Cr(VI) adsorption and removal

Cr(VI) adsorption and removal experiments were conducted under equilibrium batch conditions. A series of solutions containing predetermined concentrations of Cr(VI) was prepared by diluting a 100 mg/L standard stock solution. All experiments were performed in duplicates under ambient conditions at a controlled temperature of 25 °C. Briefly, 4 mL of 100 mg/L Cr(VI) solution (pH = 2.0) was added to individual 10 mL volumetric flasks containing 8 mg of ASNs, MSNs, NH_2_–ASNs, and NH_2_–MSNs. Samples were mixed using a magnetic stirrer at 200 rpm for ~120 min, which was enough to establish adsorption equilibrium. Then, the liquid phase of each solution obtained by filtration through a polytetrafluoroethylene (PTFE-H) membrane of a pore diameter of 0.45 μm. Cr(VI) concentrations in solutions before and after the batch experiments were measured using the DPC method, and the results were used to calculate the amount of adsorption per used sorbents (*q*_*t*_) at equilibrium.

The effect of different initial pH values was investigated in the range of 2.0–7.0 with initial Cr(VI) concentration of 100 mg/L at 25 °C. The optimum pH value for yielding the maximum adsorption capacity was adopted for the remaining experiments. The effect of initial Cr(VI) concentration was explored in the range of 1–100 mg/L. The adsorption isotherms were measured by varying initial Cr(VI) concentration from 1–100 mg/L at four different temperatures of 25, 35, 45, and 55 °C within 2 h, respectively.

Time-dependent adsorption experiments were also conducted under a kinetic batch condition. NH_2_–ASNs or NH_2_–MSNs (4 mg of each) were added to a 10 mL volumetric flask containing a 100 mg/L Cr(VI) solution. Samples were then mixed using a magnetic stirrer at 200 rpm, and filtrates were obtained after incubation for 1, 3, 5, 10, 15, 30, 60, or 120 min. The Cr(VI) concentrations in solutions before and after the kinetic batch experiments were measured using the DPC method, and the results were used to calculate *q*_*t*_ values.

Adsorption efficiency, (i.e., defined as the amount of Cr(VI) adsorbed by ASNs, MSNs, NH_2_–ASNs, and NH_2_–MSNs) and removal efficiency, (i.e., defined as the ability of non-functionalized and NH_2_–functionalized ASNs and MSNs to reduce Cr(VI) concentrations) at equilibrium, were calculated in each case using the following mathematical Eqs. 1 and 2

1 $${q}_{e}=\frac{({C}_{0}-{C}_{e})\times V}{W}$$

2 $$R=\frac{{C}_{0}-{C}_{e}}{{C}_{0}}\times 100$$

where *q*_*e*_ (mg g^−1^) is the adsorption capacity, *R* (%) is the maximum Cr(VI) removal capacity, *C*_0_ (mg L^−1^) is the initial concentration of Cr(VI), *C*_*e*_ (mg L^−1^) is the equilibrium concentration of Cr(VI) remained in the solution after the batch experiment, *V* (L) is the volume of the solution used in the batch experiment, and *W* (g) is the weight of the nanoparticles such as ASNs, MSNs, NH_2_–ASNs, and NH_2_–MSNs used as adsorbents in the batch experiments.

### Regeneration experiments

Desorption-regeneration experiment was performed 4 times for initial Cr(VI) concentration of 100 mg/L at 25 °C after Cr(VI) desorption by 0.1 M HCl solution at temperature and pH of 25 °C and 2.0. After desorption, NH_2_–ASNs and NH_2_–MSNs were filtered, dried, and re-dispersed in a 100 mL Cr(V) standard solution for 2 h incubation. The Cr(VI) concentrations in solutions before and after the incubation were determined using the DPC method. All the experiments were duplicated, and the average values of the measurements were selected for analysis and plotting while the errors of the measurements were kept within 5%.

### Data analysis of adsorption kinetics

If an adsorption rate follows *pseudo-second order kinetics* with respect to the adsorption sites of sorbents, the appropriate kinetic equation may be expressed as Eq. 3

3 $$\frac{d{q}_{t}}{dt}=k{({q}_{e}-{q}_{t})}^{2}$$

where *q*_*t*_ is the amount adsorbed per sorbents used (mg·g^−1^) at time *t* (min), *q*_*e*_ is the equilibrium adsorption capacity (mg·g^−1^), and *k* is the adsorption rate constant (g·mg^−1^·min^−1^). In general, pseudo-second order kinetics describes the kinetics of a system in which the concentration of one reactant is essentially constant, because it is present in excess, and thus, its concentration does not affect reaction rate. The reason why Eq. 3 used the term *pseudo* in our system was that the amount adsorbed on ASNs, MSNs, NH_2_–ASNs, and NH_2_–MSNs governed Eq. 3 instead of the concentration of Cr(VI) in solutions^51^. The adsorption reaction with forward (*k*_1_) and reverse (*k*_*−*1_) rates corresponding to Eq. 3 may be written as follows

4 $${\rm{Cr}}\,+\,{\rm{2AS}}\,\rightleftarrows \,{\rm{Cr}}\cdot {{\rm{AS}}}_{2}$$

where AS is a vacant adsorption site on a nanoparticle surface and Cr·AS_2_ represents a Cr(VI) ion adsorbed at two sites. Given the assumption that the adsorption process is not limited by diffusion and Eq. 4 is an elementary step, the corresponding rate of the above reaction is expressed as;

5 $$\frac{d{C}_{{\rm{Cr}}\cdot {{\rm{AS}}}_{2}}}{dt}={k}_{1}{C}_{Cr}{C}_{{\rm{AS}}}^{2}-{k}_{-1}{C}_{{\rm{Cr}}\cdot {{\rm{AS}}}_{2}}$$

where *C*_*i*_ represents to the concentration of species *i* at time *t*, *k*_*1*_ is the forward reaction rate, and *k*_*−1*_ is the reverse reaction rate. Two additional assumptions are made to simplify Eq. 5 to 3. First, as adsorption is an irreversible process, the rate of desorption, (${k}_{-1}{C}_{{\rm{Cr}}\cdot {{\rm{AS}}}_{2}}$), is relatively insignificant and can be neglected. Second, the concentration of Cr(VI) in solutions (*C*_Cr_) remains almost constant during the adsorption process^52^. Based on these assumptions, the concentrations of adsorbed species (${C}_{{\rm{Cr}}\cdot {{\rm{AS}}}_{2}}$) and vacant sites to the amounts adsorbed (${C}_{{\rm{AS}}}$) at time *t* and at equilibrium are proportional to *q*_*t*_ and *q*_*e*_ − *q*_*t*_, respectively.

6 $${C}_{{\rm{C}}{\rm{r}}\cdot {{\rm{A}}{\rm{S}}}_{2}}\propto {q}_{t}$$

7 $${C}_{{\rm{AS}}}\propto ({q}_{e}-{q}_{t})$$

Applying Eqs. 6 and 7 to Eq. 5 and subsequent integration of Eq. 5 and applying the initial condition of *q*_*t*_ = 0 at *t* = 0 resulted in a non-linear equation for *q*_*t*_ as a function of *t*.

8 $${q}_{t}(t)=\frac{{q}_{e}^{2}{k}_{2}t}{1+{q}_{e}{k}_{2}t}$$

where *k*_2_ is the pseudo-second order adsorption rate constant (g·mg^−1^·min^−1^), *q*_*t*_ is the adsorption capacity (mg·g^−1^), i.e., the amount of Cr(VI) adsorbed per mass of nanoparticles used at time *t*, and *q*_*e*_ is adsorption capacity (mg·g^−1^) at equilibrium.

A linearized form of Eq. 8 has often been used to obtain adsorption kinetics parameters using linear regression, which has been most widely used to analyze pseudo-order adsorption kinetics^51,53–55^.

9 $$\frac{t}{{q}_{t}(t)}=\frac{1}{{k}_{2}{q}_{e}^{2}}+\frac{t}{{q}_{e}}$$

Best fits to the adsorption data shown in Fig. 8A were obtained using both linear and non-linear regression using Eqs. 8 and 9, respectively. Plots of standardized residuals of the above best fits were obtained in order to assess the quality of the *good* fit (Fig. S5). Standardized residuals were calculated using the following expression

10 $${\rm{standard}}\,{\rm{residual}}\,({\rm{SR}})\,=\,\frac{{q}_{t}-{q}_{t}^{fit}}{sd}$$

where ${q}_{t}^{fit}$ (mg·g^−1^) is the amount of Cr(VI) adsorbed per mass of nanoparticles used at time *t* as predicted by best fit, and *sd* is the standard deviation of residuals (mg·g^−1^)^51^.

**Figure 1:**
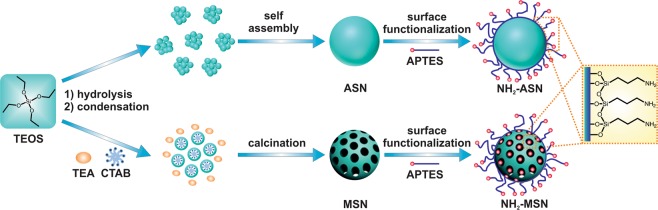
Schematic diagram of the preparation of amorphous silica nanoparticle (ASN), mesoporous silica nanoparticle (MSN), and aminopropyl-functionalized ASN and MSN.

The intraparticle diffusion kinetics, as described by the Weber-Morris model, may be expressed with the following expression

11 $${q}_{t}(t)={k}_{di}{t}^{\frac{1}{2}}+{C}_{i}$$

where *K*_*di*_ (g·mg^−1^·min^1/2^) is the rate parameter during stage *i*, which is calculated from the slope of the plot of *q*^*t*^ versus *t*^*1/2*^, and *C*_*i*_ is the intercept during stage *i*, which provides information about the thickness of the boundary layer^56,57^. Best fits to the adsorption data shown in Fig. 8B were obtained by linear regression using Eq. 11.

## Results and Discussion

### Structural analysis of ASNs and MSNs

**Figure 2:**
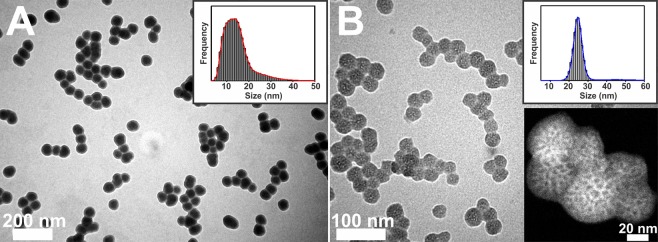
TEM micrographs of (**A**) ASNs and (**B**) MSNs. Inset images (upper right) showing histograms of measured particle sizes. The inset in (**B**) (lower right) shows an annular dark-field scanning transmission microscopy (ADF-STEM) image of MSNs, which show contrast differences due to the existence of intraparticle nanopores.

We used a method reported by Stöber and others^42–44^ to prepare amorphous silica nanoparticles (ASNs) of various sizes ranging from ~15 and ~60 nm. By using triethanolamine (TEA) and cetyltrimethylammonium chloride (CTAC) as surfactants to form sacrificial micelles, which templated pores within ASNs, we were able to produce mesoporous silica nanoparticles (MSNs) with nanoscale mesopores (a.k.a. nanopores) using a modified Stöber method^46^. Figure 1 illustrates a schematic diagram of their preparation. Figure 2 shows TEM micrographs of as-synthesized ASNs and MSNs with a relatively narrow size distribution (< ~10%). Annular dark-field scanning TEM (ADF-STEM) measurements of MSNs revealed a substantial contrast differentiation within the MSNs, which we attributed the difference of electron densities caused by the presence of nanopores (inset in Fig. 2B). These results suggest that nanopores of approximately sub-8nm diameter were present within MSNs but did not provide detailed information about the precise sizes, shapes, nor connectivities of the pores.

**Figure 3:**
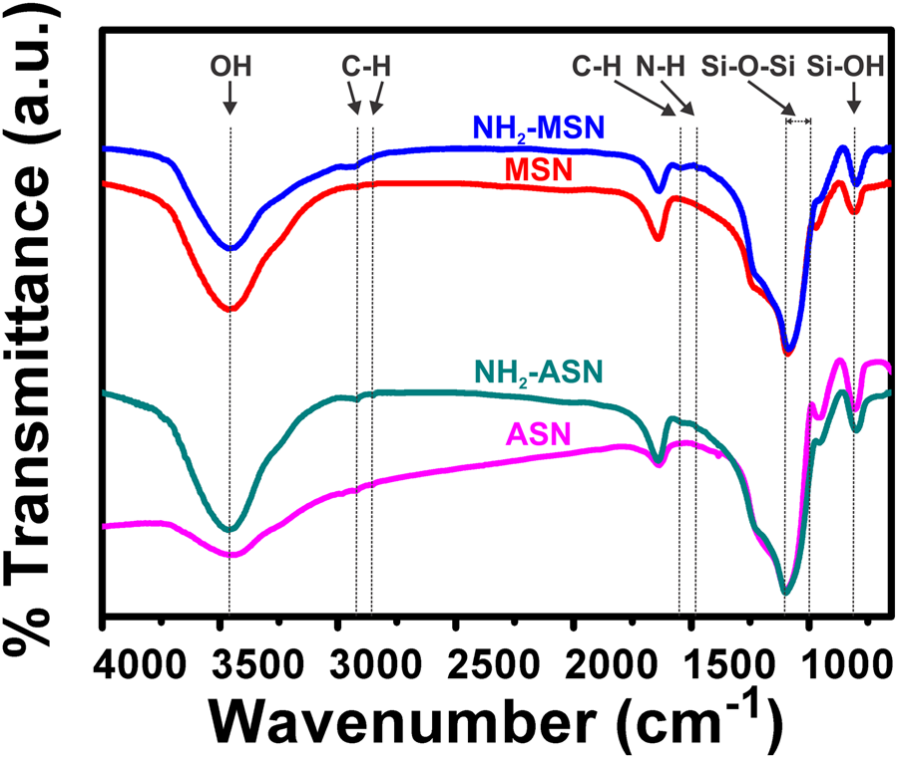
FT-IR spectra of ASNs (magenta), NH_2_–ASNs (dark cyan), MSNs (red), and NH_2_–MSNs (blue). The dotted lines indicate the characteristic IR bands of particular vibrational modes.

### FT-IR analysis of NH_2_–ASNs and NH_2_–MSNs

The ASNs and MSNs prepared were reacted with 3-aminopropyl triethoxysilane (APTES) to introduce surface amino (NH_2_–) groups immediately after their syntheses. Figure 3 shows FT-IR spectra of ASNs (magenta curve) and MSNs (red curve) before APTES functionalization exhibiting the following IR bands of silica moiety at ~3480 cm^−1^ (Si–OH), ~1100–1000 cm^−1^ (Si–O–Si), and ~920 cm^−1^ (Si–OH), which were consistent with values previously reported for silica nanoparticles synthesized by the Stöber method^58,59^. FT-IR spectra of NH_2_-functionalized ASNs (NH_2_–ASNs) and MSNs (NH_2_–MSNs) contained IR bands at ~2846–2932 cm^−1^ and ~1459 cm^−1^ (dark cyan and blue curve) that were assigned to the ν(C-H) stretching and δ(C-H) bending modes of the alkyl chain of bound APTES. The weak IR bands at ~1459 cm^−1^ of NH_2_–ASNs and of NH_2_–MSNs were assigned to the δ(N-H) bending mode of the amino group of bound APTES, respectively. These IR bands of the NH_2_–ASNs and NH_2_–MSNs were also consistent with previously reported values^60,61^.

**Figure 4:**
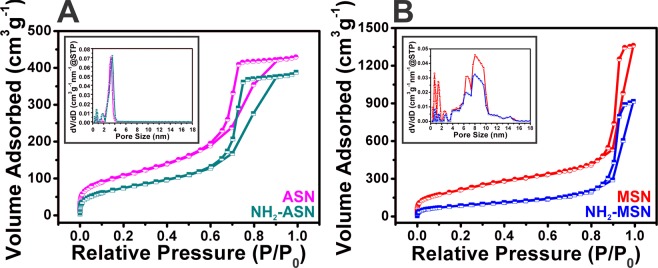
N_2_ sorption isotherms and pore size distributions of (**A**) ASNs (magenta) and NH_2_–ASNs (dark cyan) and of (**B**) MSNs (red) and NH_2_–MSNs (blue). The inset plots show the pore size distribution of (**A**) ASNs (magenta) and NH_2_–ASNs (dark cyan) and of (**B**) MSNs (red) and NH_2_–MSNs (blue).

### Specific surface area of non-functionalized and NH_2_–functionalized ASNs and MSNs

Low temperature N_2_ adsorption-desorption isotherms of ASNs, MSNs, and NH_2_–ASNs and NH_2_–MSNs were obtained to determine their specific surface areas and porosities. Figure 4 shows N_2_ sorption isotherms and pore size distributions of ASNs, NH_2_–ASNs, MSNs, and NH_2_–MSNs. The shapes of all four isotherms were similar to that of the characteristic type IV isotherm described in the IUPAC classification^62,63^. In particular, the isotherms of the ASNs and NH_2_–ASNs exhibited distinctive type H2 hysteresis (IUPAC classification) with an extended loop indicating the existence of disordered pores in the pressure (*P*/*P*_0_) range between 0.6 and 1. However, the hysteresis loop of isotherms of MSNs and NH_2_–MSNs was similar to type H1 hysteresis (IUPAC classification), which is commonly observed for mesoporous silica materials with well-defined cylindrical pores^64,65^ within the same pressure (*P*/*P*_0_) range. The pore size distributions of ASNs and NH_2_–ASNs (inset plots in Fig. 4A), as determined by the Brunauer-Emmett-Teller (BET)^66^ and Barrett-Joyner-Halenda (BJH)^67^ analysis, showed a major peak corresponding to their most probable size at ~3 nm. On the other hand, the pore size distributions of MSNs and NH_2_–MSNs (inset in Fig. 4B) revealed additional peaks, which suggested the presence of pores of different types slightly larger than ~3 nm. These results suggest that the major peaks observed in the pore size distributions of MSNs and NH_2_–MSNs were probably caused by void interstices formed by the external surfaces of the aggregated nanoparticles and that the additional peaks observed in those of MSNs and NH_2_–MSNs were caused by disordered nanopores within the nanoparticles.

**Figure 5:**
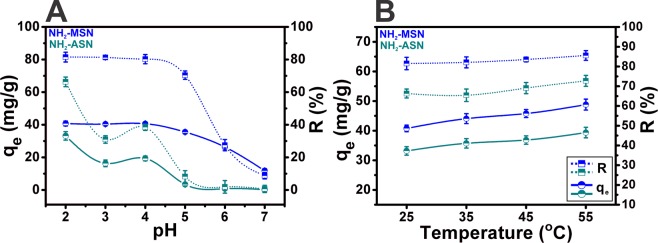
Effect of pH and temperature on Cr(VI) adsorption and removal. The adsorption capacity (*q*_*e*_) and removal efficiency (*R*) of NH_2_–ASNs and NH_2_–MSNs as a function of solution pH and temperature.

The specific surface areas (*S*_*BET*_) and pore sizes of ASNs, MSNs, NH_2_–ASNs, and NH_2_–MSNs were determined from their low temperature N_2_ adsorption-desorption isotherms using the BET and BJH models. Table 1 summarizes the results. Measured specific surface areas of ASNs, NH_2_–ASNs, MSNs, and NH_2_–MSNs were 387.0, 272.9, 792.1, and 517.4 m^2^·g^−1^, respectively. Notably, the specific surface areas of MSNs and NH_2_–MSNs were nearly twice those of ASNs and NH_2_–ASNs, which suggested the presence of nanopores.

**Table 1:** Sizes, specific surface areas (*S*_*BET*_), pore volumes, pore sizes, and functional group loadings of ASNs, NH_2_–ASNs, MSNs, and NH_2_–MSNs as determined by TEM^a^ measurements, N_2_ sorption isotherms (BET^b^ and BJH^c^ models), and TGA/DTA^d^ measurements.

Material	Particle diameter (nm)^a^	Specific surface area (m^2^ g^−1^)^b^	Specific surface area (m^2^ g^−1^)^c^	Pore volume (cm^3^ g^−1^)^c^	Pore diameter (nm)^c^	Functional group loading (mmol g^−1^)^d^
ASN	15 ± 1	387.0	281.9	0.66	3.43	−
NH_2_-ASN	15 ± 1	272.9	189.9	0.60	4.40	0.25
MSN	26 ± 2	792.1	517.4	2.11	5.32	−
NH_2_-MSN	26 ± 2	517.4	213.5	1.42	8.94	0.81

It has been reported that the hysteresis exhibited by the sorption isotherms of ASNs and NH_2_–ASNs is associated with capillary condensation in porous structures^62,68^. Because ASNs and NH_2_–ASNs can be modeled as hard spheres comprised of bulk SiO_2_, the arrangements of aggregated ASNs and NH_2_–ASNs and the interfacial gaps between particles also suggest that the hysteresis loop in their isotherms reflects porosity. Therefore, the *S*_*BET*_ values of ASNs and NH_2_–ASNs are likely to be the result of contributions, by the geometric surface areas of ASNs and NH_2_–ASNs that are presumed to behave like hard nanospheres, and the surface areas of the porous architecture formed by the aggregations of these nanoparticles. On the other hand, the *S*_*BET*_ values of mesoporous materials are equal to the sum of geometric surface, and micro- and meso-pore surface^28,69^.

The geometric specific surface areas (*S*_*ext*_) of nonporous spherical silica particles are inversely proportional to their diameter ‘*d*’ as described by the following mathematical expression 12^28,70^, where ρ is the density of bulk silica (SiO_2_).

12 $${S}_{ext}=\frac{{\rm{surface}}\,{\rm{area}}\,{\rm{of}}\,{\rm{a}}\,{\rm{particle}}}{{\rm{density}}\,\times \,{\rm{volume}}\,{\rm{of}}\,{\rm{a}}\,{\rm{particle}}}=\frac{4\pi {(d/2)}^{2}}{{\rho }_{{{\rm{SiO}}}_{2}}\times \frac{4}{3}\pi {(d/2)}^{3}}=\frac{6}{{\rho }_{{{\rm{SiO}}}_{2}}\times d}$$

Thus, *S*_*ext*_ can be calculated from the *d* values of the silica nanoparticles as determined by TEM measurements. Based on assumptions made for the case of ASNs and NH_2_–ASNs, the *S*_*BET*_ values of ASNs and NH_2_–ASNs were considered to be approximately equal to the sum of *S*_*ext*_ and the surface area contributions of inner pores and resulting porous structures formed by the external surfaces of the aggregated nanoparticles (*S*_*t*_) according to the following expression 13.

13 $${S}_{BET}={S}_{t}+{S}_{ext}$$

This approach has proven to be useful for understanding the nature of the specific surface areas of solid powders comprised of aggregated nanoparticles because the walls of their interstices are presumably formed by the external surface of the nanoparticles^48^. In the case of MSNs and NH_2_–MSNs, it is reasonable to assume that nanopore specific surface area (*S*_*np*_) contributes to *S*_*BET*_, as described by the following expression 14.

14 $${S}_{BET}={S}_{t}+{S}_{ext}+{S}_{np}$$

In the case of 26 nm diameter MSNs and NH_2_–MSNs, interstitial environments are probably similar to those of comparably sized ASNs and NH_2_–ASNs. Therefore, it is reasonable to assume that the *S*_*t*_ values of 26 nm diameter MSNs and NH_2_–MSNs are similar to those of 26 nm diameter ASNs and NH_2_–ASNs, respectively. Size-corrected specific surface areas (*S’*_*BET*_) of 26 nm diameter ASNs and NH_2_–ASNs were extrapolated from a plot of *S*_*BET*_ versus ASN size (see Fig. S1) and consequently, the corresponding *S*_*t*_ of 26 nm diameter MSNs and NH_2_–MSNs were estimated by the *S*_*ext*_ of 26 nm diameter MSNs and NH_2_–MSNs from extrapolated *S’*_*BET*_ values using the expression 13. In addition, the *S*_*np*_ of 26 nm diameter MSNs and NH_2_–MSNs were obtained from the expression 14 using the calculated *S’*_*BET*_ and *S*_*ext*_ values.

Table S1 summarizes various specific surface area types of ASNs, NH_2_–ASNs, MSNs, and NH_2_–MSNs. The *S*_*np*_ values of MSNs and NH_2_–MSNs were 519.0 and 358.4 m^2^·g^−1^, respectively. Comparison of the *S*_*np*_ and *S*_*BET*_ values of MSNs and NH_2_–MSNs showed that the surface area contributions due to the presence of the nanopores were>65% of the total specific surface area (*S*_*BET*_). Accordingly, the geometric scaling approach enabled the different types of specific surface area of MSNs and NH_2_–MSNs to be evaluated, and the results obtained demonstrated the important roles of nanopores play in the adsorption of Cr(VI).

### TGA analysis of ASNs, MSNs, NH_2_–ASNs, and NH_2_–MSNs

Amounts of amino groups on the surface of NH_2_–ASNs and NH_2_–MSNs were estimated by thermogravimetric analysis/differential thermal analysis (TGA/DTA). Figure S2 shows TGA/DTA curves of ASNs (magenta curve), NH_2_–ASNs (dark cyan curve), MSNs (red curve), and NH_2_–MSNs (blue curve) showing weight losses as a function of temperature. Based on TGA/DTA measurements, the functional group loading values of NH_2_–ASNs and NH_2_–MSNs were determined to be ~0.25 and ~0.81 mmol·g^−1^, respectively. Table S2 summarizes weight losses and functional group loadings of ASNs, NH_2_–ASNs, MSNs, and NH_2_–MSNs. Note that the functional group loading on NH_2_–MSNs was more than three times greater than on NH_2_–ASNs, which we attribute the presence of nanopores.

### Effects of pH and temperature on Cr(VI) adsorption and removal from aqueous solutions using ASNs, MSNs, NH_2_–ASNs, and NH_2_–MSNs

ASNs and MSNs exhibited an extremely lower Cr(VI) adsorption (i.e., their adsorption capacity (*q*_*e*_) obtained using Eq. 1 less than ~4% of those of NH_2_–ASNs, and NH_2_–MSNs shown in Table 2 and Fig. 8A), which was considered to be relatively insignificant. Thus, only the Cr(VI) adsorption and removal of NH_2_–ASNs, and NH_2_–MSNs were investigated for most of the batch experiments. Figure 5 shows the effects of pH and temperature on Cr(VI) adsorption and removal from aqueous solutions in the pH and temperature range of 2.0–7.0 and 25–55 °C, respectively. A few aspects of the results are worth addressing. First, the adsorption capacity (*q*_*e*_), defined in Eq. 1, of NH_2_–ASNs and NH_2_–MSNs increased from 33.2 to 39.4 and 40.8 to 44.8 mg·g^−1^ while the removal efficiency (*R*), defined in Eq. 2, slightly increased from 66.3 to 72.7 and 81.5 to 85.6% with the increasing of temperature, respectively. Although the amounts of increase were not high, they were consistent with the hypothesis that raising the temperature from 25 to 55 °C marginally favors the adsorption of Cr(VI). Also, the increases in the adsorption capacity of NH_2_–ASNs and NH_2_–MSNs with the increasing temperature implied the endothermic nature of the adsorption process. Second, the *q*_*e*_ of NH_2_–ASNs and NH_2_–MSNs improved from 0.2 to 33.2 and 11.8 and 40.8 mg·g^−1^, and the *R* from 0.6 to 66.3 and 8.8 to 82.2% significantly by decreasing the pH of the solution from 7.0 to 2.0, respectively. Hence, our results indicated that a higher adsorption capacity was obtained at a lower pH.

**Table 2:** Adsorption and removal efficiency parameters of ASNs, NH_2_–ASNs, MSNs, and NH_2_–MSNs as determined by Cr(VI) adsorption batch experiments. Cr(VI) adsorption efficiencies^a^ were obtained from spectrophotometric measurements using the DPC method. Cr(VI) removal efficiencies^b^ were calculated using Eqs. 1 and 2. Total Cr removal^c^ was estimated based on the amount of Cr(VI) and Cr(III) adsorbed on nanoparticle surfaces. Cr(VI) reduction^d^ was estimated by considering the amount of Cr(III) in aqueous solutions and on the surfaces of nanoparticles.

Material	Cr(VI) adsorption efficiency^a^ (mg g^−1^)	Cr(VI) removal efficiency^b^ (%)	Total Cr removal^c^ (mg g^−1^)	Cr(VI) reduction^d^ (mg g^−1^)
ASN	0.4	0.7	—	—
MSN	1.3	2.4	—	—
NH_2_-ASN	34.0	61.9	32.6	11.5
NH_2_-MSN	42.2	76.8	39.8	34.7

**Figure 6:**
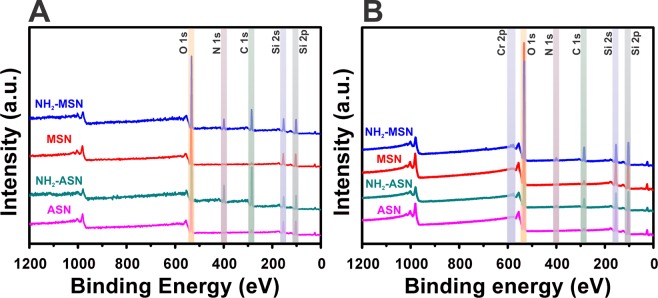
Survey XPS spectra of ASNs (magenta), NH_2_–ASNs (dark cyan), MSNs (red), and NH_2_–MSNs (blue) before (**A**) and after (**B**) Cr adsorption. Note the presence of C 1s and N 1s peaks in the survey spectra in (**A** and **B**), which confirmed NH_2_–functionalization of ASNs (NH_2_–ASNs) and MSNs (NH_2_–MSNs), and the Cr 2p peaks in **B** after Cr(VI) adsorption.

The effect of pH can be rationalized by considering the surface charge of the nanoparticle adsorbents and the degree of ionization of Cr(VI) species. In general, Cr(VI) ions exist in several anionic forms such as HCrO_4_^−^, CrO_4_^2−^, HCr_2_O_7_^−^, and Cr_2_O_7_^2−^ in aqueous solution depending on the solution pH and concentration^3^. H_2_CrO_4_ exists primarily at strong acidic pH (< ~2) and HCrO_4_^−^ and Cr_2_O_7_^2−^ are usually detected at pH range of 2.0–6.0. When the pH increases to 7.0 and above, CrO_4_^2−^ is likely to be the primary ionic species. Considering that the pK_a_ of the NH_2_–groups lies within the pH range 9–10, the surfaces of NH_2_–ASNs and NH_2_–MSNs are likely to be composed of ammonium (NH_3_^+^-) and silanol (-Si-O-H) moieties and confer nanoparticle surfaces a net positive charge under acidic conditions^71^. Figure S3 shows measurements of pH at the point of zero charge (pH_pzc_) on the surface of NH_2_-functionalized ASNs and MSNs. The values of pH_pzc_ of NH_2_–ASNs and NH_2_–MSNs were determined to be 8.5 and 8.4, respectively. These results provide compelling evidence that the surface of NH_2_–ASNs and NH_2_–MSNs is positively charged for pH below the value of pH_pzc_. Since Cr(VI) species formed under these conditions are likely to be anionic HCrO_4_^−^ and Cr_2_O_7_^2−^, these results warrant electrostatic attraction of the anionic Cr(VI) species. However, with increasing pH, the amount of NH_2_–groups to be protonated is likely dropped. At the same time, the concentration of hydroxyl (OH^−^) ion increased and electrostatically competed against anionic Cr(VI) species, leading to a decline of Cr(VI) adsorption and removal efficiency. In summary, a very low solution pH was required to achieve high protonation of the surface NH_2_–groups and provide the necessary electrostatic attraction of anionic Cr(VI) species. Consequently, pH = 2.0 was selected as the optimal pH of Cr(VI) aqueous solution for the following batch experiments at 25 °C.

### Cr(VI) adsorption and removal efficiency of ASNs, MSNs, NH_2_–ASNs, and NH_2_–MSNs

A series of batch experiments on Cr(VI) adsorption and removal was carried out at the optimal pH and temperature of 2.0 and 25 °C, respectively. Table 2 summarizes the adsorption and removal efficiency parameters of ASNs, MSNs, NH_2_–ASNs, and NH_2_–MSNs by the Cr(VI) adsorption batch experiments. Notably, the adsorption efficiencies (*q*_*e*_) of NH_2_–ASNs and NH_2_–MSNs were ~40 to 80 times greater than those of ASNs and MSNs, which confirmed the impact of NH_2_–functionalization on the Cr(VI) adsorption efficiency^27,39^. Maximum Cr(VI) removal capacity (*R*) represents the ability of an adsorbent to remove Cr(VI) from aqueous environments. The maximum Cr(VI) removal capacities of NH_2_–ASNs and NH_2_–MSNs were determined to be 61.9 and 76.8%, respectively. These values were comparable or marginally better than those reported for functionalized silica nanoparticles^72,73^, mesoporous silica^6,39,48,74–76^, and other hybrid multifunctional nanoparticles^77,78^ designed for Cr(VI) removal. Notably, the maximum Cr(VI) removal capacity of NH_2_–MSNs was 76.8%, which was 1.2 times higher than the 61.9% of NH_2_–ASNs. Thus, our results suggest that a combination of a high density of nanopores within silica nanoparticles and high surface coverage by functional amine groups enhance both adsorption and removal efficiencies of Cr(VI).

### XPS analysis of Cr(VI) adsorption on ASNs, MSNs, NH_2_–ASNs, and NH_2_–MSNs

Figure 6 shows survey XPS spectra of ASNs, MSNs, NH_2_–ASNs, and NH_2_–MSNs before and after Cr(VI) adsorption. The survey XPS spectra in Fig. 6A, which were obtained over the full energy range confirm the existence of common elements such as O and Si before Cr(VI) adsorption. Note that the presence of C 1 s and N 1 s peaks in the spectra of NH_2_–ASNs and NH_2_–MSNs in Fig. 6A confirmed the presence of surface NH_2_ moieties. High resolution XPS scans of NH_2_–ASNs and NH_2_–MSNs in Fig. S4 revealed the following binding energies (BEs): a C 1 s peak at 284.7–284.9 eV, a N 1 s peak at 399.0–399.1 eV, a O 1 s peak at 532.2–532.3 eV, and a Si 2p peak at 101.9–102.3 eV. Comparison of O 1 s spectra of NH_2_–ASNs and NH_2_–MSNs in Fig. S4 shows that their oxygen BEs were similar to those of bound oxygen in bulk silicon di- and sub-oxides, which typically appear at 532.0–533.0 eV. It is worth mention that the Si 2p spectra of NH_2_–MSNs showed a single peak at 102.3 eV, which was marginally higher than that of NH_2_–ASNs at 101.9 eV. Considering the fact that the characteristic Si 2p BE peaks of elementary Si and SiO_2_ reported in the literature are at 99.5 and 103.5 eV^79^, respectively, our results imply that silicon in the surface layer of NH_2_–ASNs and NH_2_–MSNs may exist as intermediate sub-oxide phases (SiO_x_, 0 < x ≤ 1) rather than the fully oxidized SiO_2_^79,80^ due to a greater presence of elementary non-oxidized Si in the surface layers of NH_2_–ASNs and NH_2_–MSNs. Furthermore, these values were consistent with those reported for functionalized silica nanoparticles^39,81^, and mesoporous silica^6,46^ systems.

**Figure 7:**
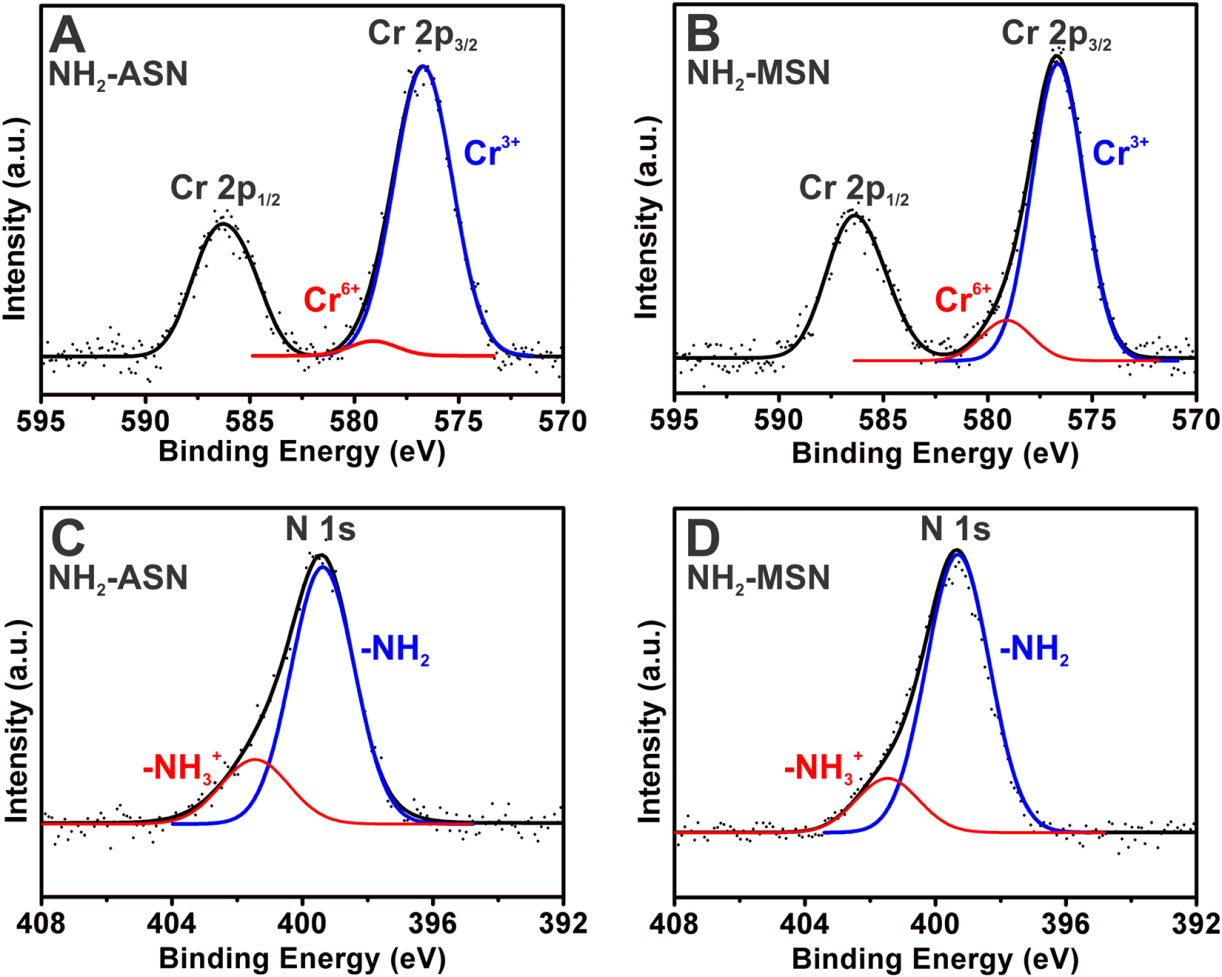
High resolution XPS spectra of Cr 2p (**A** and **B**) and N 1s (**C** and **D**) of NH_2_–ASNs and NH_2_–MSNs after Cr(VI) adsorption. Dotted black curves were obtained from XPS measurements, and solid black curves are best fits obtained by Lorentzian-Gaussian deconvolution. Blue and red curves obtained by the deconvolution of (**A** and **B**) were assigned to Cr(III) and Cr(VI), respectively. Blue and red curves of (**C** and **D**) were assigned to neutral NH_2_- and cationic NH_3_^+^- moieties on the surfaces of NH_2_–ASNs and NH_2_–MSNs after Cr(VI) adsorption, respectively.

The presence of Cr 2p peaks in the survey XPS spectra of NH_2_–ASNs, and NH_2_–MSNs (Fig. 6B) confirmed the surface adsorption of chromium. Figure 7 shows high resolution Cr 2p and N 1 s XPS spectra of the Cr adsorbed NH_2_–ASNs and NH_2_–MSNs. The two asymmetric peaks shown in Fig. 7A,B were assigned to Cr 2p_1/2_ and Cr 2p_3/2_ orbitals. Deconvolution of the corresponding Cr 2p_2/3_ spectra in Fig. 7A,B showed two symmetric peaks that were best fitted by Lorentzian-Gaussian functions. The peak (blue) centered at ~576.7 eV was assigned to Cr(III) species, while peak (red) centered at ~579.1 eV was attributed to Cr(VI) based on previously reported values^6,82^. The relative ratios of Cr(III) to Cr(VI) on the surface of the NH_2_–ASNs and NH_2_–MSNs (Cr(III)/Cr(VI)) were ~24.7 and ~7.5, respectively, and were obtained from the areas of the Lorentzian-Gaussian curves (indicated by blue and red curves in Fig. 7). These values imply that most of the Cr adsorbed was in oxidation state III rather than VI. Therefore, the amount of Cr(VI) reduced to Cr(III) was estimated from the values in Table 2. The quantity of total Cr removal in Table 2 corresponds to the amount of chromium ions, including Cr(VI) and Cr(III), eliminated from the solutions after the batch adsorption experiments. They were quantitatively determined by inductively coupled plasma optical emission spectroscopy (ICP OES) in addition to the DPC method. Note that the values of total Cr removal were smaller than that of Cr(VI) adsorption capacity in Table 2. We hypothesize that this may be because a significant proportion of adsorbed Cr(VI) ions was reduced to Cr(III), and a fraction of adsorbed Cr(III) ions was released back to the aqueous solutions^6^. Based on the hypothesis, the values of Cr(VI) reduction in Table 2 were obtained by determining both the amount of Cr(III) ions in solution and the amount of Cr(III) adsorbed on the surface of NH_2_–ASNs and NH_2_–MSNs measured by high resolution XPS.

**Figure 8:**
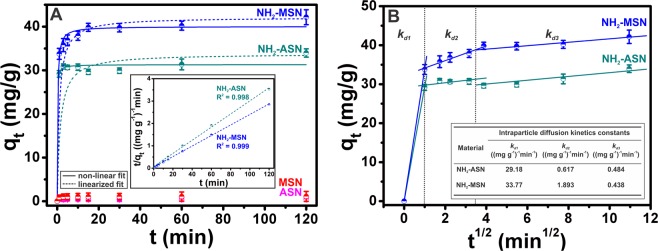
(**A**) Cr(VI) adsorption kinetics of ASNs (magenta), MSNs (red), NH_2_–ASNs (dark cyan), and NH_2_–MSNs (blue) as determined by assuming pseudo-second order adsorption kinetics. Best fits to data were successfully obtained using linearized and non-linear fits. (**B**) Cr(VI) adsorption kinetics of NH_2_–ASNs and NH_2_–MSNs as determined using the intraparticle diffusion kinetics model. Best fits to data were successfully obtained in time regions, which indicated Cr(VI) adsorption was a multi-step process.

Our high resolution XPS results in Fig. 7, the pH_pzc_ vaules, and the results summarized in Table 2 suggest that adsorption is initiated by electrostatic attraction between anionic Cr(VI) species and surface ammonium groups followed by the reduction of most Cr(VI) to Cr(III). Fellenz *et al*. reported that the Cr(III) to Cr(VI) ratio of chromium adsorbed on aminopropyl-functionalized MCM-41 sorbents as determined by XPS was close to 1 after Cr(VI) adsorption from aqueous environments at pH ~2.0^6^. They also analyzed the XPS spectra of the same samples in the region of N 1 s and determined that N 1 s BEs of ~400.0 and ~402.0 eV corresponded to cationic ammonium (–NH_3_^+^) and neutral amino (–NH_2_) moieties, respectively, which concurred with previously reported values^83^. Figure 7C,D show deconvoluted N 1 s XPS spectra of Cr adsorbed NH_2_–ASNs and NH_2_–MSNs were composed of two peaks located at ~399.3 and ~401.5 eV, which were assigned to –NH_3_^+^ and –NH_2_ groups, respectively. In the present study, the relative ratios of –NH_2_ to –NH_3_^+^ groups on the Cr adsorbed NH_2_–ASNs and NH_2_–MSNs as determined from areas of the Lorentzian-Gaussian curves, were ~3.7 and ~5.2, respectively. These values suggest that –NH_2_ surface coverage was considerably higher than that of –NH_3_^+^ group coverage, which contradicts expectation because at a pH of ~2.0 –NH_3_^+^ groups should predominate^71^. We offer the following explanation for this observation. First, anionic HCrO_4_^−^ and Cr_2_O_7_^2−^ species electrostatically interact and bind to surface cationic –NH_3_^+^ of NH_2_–ASNs and NH_2_–MSNs, and subsequently Cr(VI) is reduced to less toxic Cr(III), and either H^+^ from NH_3_^+^–groups is likely released to medium, or some of adsorbed Cr(III) are desorbed and released to the aqueous solution to maintain electrostatic charge balance. This explanation is reasonably consistent with the Cr(III) to Cr(VI) ratios shown in Fig. 7, although these ratios were slightly higher than previously reported values^6,39^.

### Cr(VI) adsorption kinetics of ASNs, MSNs, NH_2_–ASNs, and NH_2_–MSNs

The Cr(VI) adsorption kinetics of ASNs, MSNs, NH_2_–ASNs, and NH_2_–MSNs are shown in Fig. 8. Figure 8A shows that >95% of Cr(VI) was rapidly adsorbed by NH_2_–ASNs and by NH_2_–MSNs during the first few minutes of experiments and that the remainder of the Cr(VI) was adsorbed slowly. The Cr(VI) adsorption on NH_2_–ASNs and NH_2_–MSNs exhibited a saturation behavior when the contact time reached 120 min, which suggests not enough adsorption sites for anionic Cr(VI) species were available. As more anionic Cr(VI) species were adsorbed on NH_2_–ASNs and NH_2_–MSNs, more electrostatic repulsion between negatively charged Cr(VI) species likely resulted in decreasing the removal efficiency. Although the Cr(VI) adsorption kinetics of ASNs and MSNs were similar to the trend of NH_2_–ASNs, and NH_2_–MSNs, they exhibited an extremely lower adsorption behavior with their *q*_*e*_ values less than ~4% of those of NH_2_–ASNs, and NH_2_–MSNs. Because the adsorption capacities of ASNs and MSNs were considered to be relatively insignificant, only the results of NH_2_–ASNs, and NH_2_–MSNs were analyzed by obtaining best fit to data using *a linearized form* of the *pseudo-first order model*^84,85^, for which *log(q*_*e*_*-q*_*t*_) was plotted versus *t*, and *a linearized form* of the *pseudo-second order model*^86,87^, for which *q*_*t*_*/t* was plotted versus *t/q*_*e*_. These models are frequently used to describe various adsorption processes involving widely different adsorbents and adsorbates^52,88^. The validity of each model was confirmed using correlation coefficients (*R*^2^) and by checking agreement between experimental and fitted value of *q*_*e*_. The inset plot in Fig. 8A shows that applying the linearized form of the pseudo-second order model to our data resulted in much higher correlation coefficients (*R*^2^ > 0.99) than pseudo-first model (*R*^2^ < 0.86). Surprisingly, corresponding plots with correlation coefficients (*R*^2^ > 0.99) of the Cr(VI) adsorption kinetics of NH_2_–ASNs and NH_2_–MSNs (dashed curves in Fig. 8A) did not produce *good* fits using the *linearized fitting process* particularly for time between 0 and 20 min. Instead, using *nonlinear regression* and a pseudo-second order model resulted in much improved fit (solid curves in Fig. 8A) especially for time in the same window. As regards *q*_*e*_, reasonably good agreement was also obtained between calculated (*q*_*e,fit*_) and experimental (*q*_*e,exp*_) values for NH_2_–ASNs and NH_2_–MSNs using a pseudo-second order model. Table 3 summarizes the kinetic parameters of Cr(VI) adsorption kinetics on NH_2_–ASNs and NH_2_–MSNs as determined by best fits with the pseudo-second order model using nonlinear regression. It is worth mention that the *q*_*e,fit*_ value of NH_2_–MSNs obtained under equilibrium condition was 40.01 mg·g^−1^ and this was ~1.3 times greater than that of NH_2_–ASNs. On the other hand, the pseudo-second order rate constant (*k*_2_) of NH_2_–MSNs was 0.111 (mg·g^−1^)^−1^·min^−1^, which was barely one-fourth of that of NH_2_–ASNs.

**Table 3:** Kinetic parameters of Cr(VI) adsorption on NH_2_–ASNs and NH_2_–MSNs determined from best fits to the pseudo-second order model shown in Fig. 8A using linearized^a^ and nonlinear^b^ fits.

Material	*q*_*e,exp*_ (mg g^−1^)	Pseudo-2^nd^ order model (linearized fit)^a^			Pseudo-2^nd^ order model (nonlinear fit)^b^	
		*k*_2_ ((mg g^−1^)^−1^ min^−1^)	*q*_*e,fit*_ (mg g^−1^)	*R*^2^	*k*_2_ ((mg g^−1^)^−1^ min^−1^)	*q*_*e,fit*_ (mg g^−1^)
NH_2_-ASN	33.97	0.020	33.78	0.998	0.430	31.28
NH_2_-MSN	42.16	0.023	41.13	0.999	0.111	40.01

The reason why the *well-accepted* linearized form of the pseudo-second order model did not lead to a *good* fit is unclear. This observation implies that the Cr(VI) adsorption process might violate one of the important assumptions of the pseudo-second order model, namely that, 1) the adsorption process is irreversible with no desorption, 2) the concentration of Cr(VI) in medium remains essentially constant, at least during the early adsorption period, and 3) the adsorption process is not limited by diffusion^52^. Xiao *et al*. investigated the reasons why they obtained spuriously high value of correlation coefficients (*R*^2^ ~ 1) even when a linearized form of the pseudo-second order model resulted in questionable fits^51^. They concluded that if the pseudo-second order model truly represents experimental data, the nonlinear form should be used instead and that the result be verified using a standardized residual (SR) plot to determine whether residuals are randomly determined and their standard deviations are close to zero^51^. Figure S5 shows that nonlinear fitting delivered the best overall fits with relatively low standardized residuals randomly distributed near zero (i.e., standardized deviations near zero), which was not the case for linearized fitting, and is consistent with a suggestion by Xiao *et al*.^51^. Therefore, our results show that pseudo-second order adsorption kinetics appropriately describe the adsorption of Cr(VI) by NH_2_–ASNs and NH_2_–MSNs. In addition, our results also suggest that Cr(VI) adsorption is the result of chemisorption by NH_2_–ASNs or NH_2_–MSNs via electrostatic interaction, which is also consistent with our XPS results.

The intraparticle diffusion kinetics based on the Weber-Morris model was used to investigate Cr adsorption process to obtain insight of the mechanism involved. Figure 8B shows the adsorption kinetics of Cr(VI) on NH_2_–ASNs and NH_2_–MSNs in three separate time windows and best fits to data resulted in three straight lines for plots of *q*_*t*_ versus *t*^*1/*2^. These linearities imply adsorption probably involves three steps, and the slopes of these plots may determine adsorption rate during these steps. Inset table in Fig. 8B presented the values of the rate constant *k*_*d1*_, *k*_*d*2_, and *k*_*d3*_ involving three steps. Several aspects of processes based on the intraparticle diffusion kinetics are worth addressing. First, the *k*_*d1*_ value of NH_2_–MSNs was slightly larger than that of NH_2_–ASNs probably due to their higher specific surface area. However, the rate constant *k*_*d*2_ and *k*_*d3*_ of NH_2_–ASNs and NH_2_–MSNs were not significantly different. Second, a comparison of the rate constants (i.e., *k*_*d1*_≫ *k*_*d*2_ > *k*_*d3*_) revealed that the second and third steps of Cr(VI) adsorptions on NH_2_–ASNs and NH_2_–MSNs were slower than the first and that the rate constants *k*_*d*2_ and *k*_*d3*_ were similar to those obtained by nonlinear fitting using the pseudo-second order model. This analysis suggests the second and third steps were rate-limiting. Third, the results of our analysis based on the intraparticle diffusion kinetics is reasonably consistent with the idea that the concentration of Cr(VI) in solutions gradually decreased with time without significant desorption of Cr(VI) from the surface of NH_2_–ASNs and NH_2_–MSNs. Above all, our analyses show that the intraparticle diffusion kinetic model could interpret the mechanism of adsorption to a reasonable extent.

A few questions remain concerning our analysis of Cr(VI) adsorption kinetics. First, our results provide limited data of *q*_*t*_ within the early time window (*t* ≪ 1 min) because measuring the amount of Cr(VI) adsorption after ≤ 1 min by the DPC methods was difficult and would probably result in unreliable measurements. Therefore, the lower bounds of *k*_*d1*_ rate constants were estimated using best fits with data. Based on these estimates, we concluded that the first step, which was completed within ≤ 1 min, is probably related to the instantaneous adsorption of Cr(VI) onto the external surfaces of NH_2_–ASNs and NH_2_–MSNs. Since Cr(VI) concentration in aqueous environments is relatively high during the initial step, a large concentration gradient may drive the diffusion of Cr(VI) species to the surfaces of NH_2_–ASNs and NH_2_–MSNs. It is worth recalling that the lower bound rate constant *k*_*d1*_ was ~6 times greater than those of Cr(VI) physisorption on Fe_3_O_4_ nanoparticles hybridized with carbonaceous materials^88^ and on unfunctionalized mesoporous silica^38^. Though we cannot say definitively that direct comparison between our system and these systems was valid, a sudden increment during the initial step is reminiscent of an enhancement effect by specific functional groups, such as –NH_2_ or –NH_3_^+^ groups, which likely accelerate the initial step of Cr(VI) chemisorption process by lowering the kinetic energy barrier. Second, it may be inadvisable to separate the second and third steps because *k*_*d*2_ and *k*_*d3*_ values were of the same order of magnitude as those obtained by nonlinear fitting using the pseudo-second order model. However, these rate-limiting steps may well be involved in an interplay between intraparticle diffusion and equilibration processes at extremely low Cr(VI) concentrations. We do not think it is likely that significant desorption of Cr(VI) occurred because most of the Cr(VI) adsorbed was reduced to Cr(III), as determined by XPS, and little Cr(VI) remained on NH_2_-ASNs and NH_2_-MSNs during the second and third steps.

### Effect of temperature and adsorption isotherm of NH_2_–ASNs and NH_2_–MSNs

**Figure 9:**
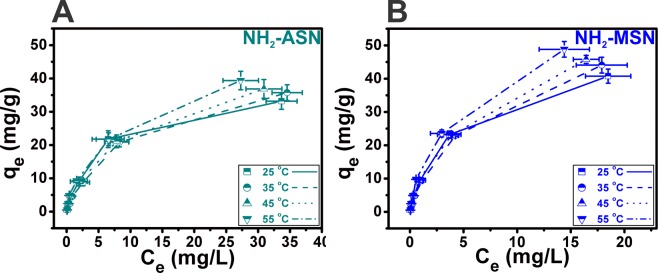
Cr(VI) adsorption isotherms of (**A**) NH_2_–ASNs and (**B**) NH_2_–MSNs as a function of temperature.

Figure 9 shows the correlation curves between the equilibrium adsorption capacity (*q*_*e*_) and equilibrium concentration (*C*_*e*_) of Cr(VI) under four different temperatures at pH of 2.0. The equilibrium adsorption capacity of NH_2_–ASNs and NH_2_–MSNs increased with the increasing of temperature, which was consistent with the results shown in Fig. 5B. To understand the relationship between *q*_*e*_ and *C*_*e*_ under equilibrium conditions, the adsorption isotherms of NH_2_–ASNs and NH_2_–MSNs were analyzed by obtaining best fits to data using the extensively used Langmuir, Freundlich, Temkin, and Dubinin-Radushkevich (D-R) models^89^.

Langmuir model, which was primarily designed to describe gas-solid phase adsorption processes, has been often used to quantify the adsorption capacity of various adsorbents in solutions^90^. The Langmuir isotherm was expressed in the following *nonlinear* and *linearized form*

15 $${q}_{e}=\frac{{q}_{m}{K}_{L}{C}_{e}}{{K}_{L}{C}_{e}+1}$$

16 $$\frac{{C}_{e}}{{q}_{e}}=\frac{1}{{q}_{m}}{C}_{e}+\frac{1}{{q}_{m}{K}_{L}}$$

where *K*_*L*_ (L·mg^−1^) and *q*_*m*_ (mg·g^−1^) is the Langmuir isotherm constant and the corresponding saturation adsorption capacity, respectively.

Freundlich model has been used to characterize adsorption processes that occur on heterogeneous surfaces^91^. Its isotherm provides useful information on the surface heterogeneity, the exponential distribution of active sites, and their energetics. The Freundlich isotherm was expressed in the following *nonlinear* and *linearized form*

17 $${q}_{e}={K}_{F}{C}_{e}^{\frac{1}{n}}$$

18 $$\mathrm{ln}\,{q}_{m}=\frac{1}{n}\,\mathrm{ln}\,{C}_{e}+\,\mathrm{ln}\,{K}_{F}$$

where *K*_*F*_ (mg·g^−1^) is the Freundlich isotherm constant that is an approximate indicator of adsorption capacity, while *1/n* is the measure of adsorption intensity.

Temkin model takes into account the effects of adsorbate-adsorbate interactions. It assumes that the heat of adsorption of all molecules in the layer decreases *linearly* rather than *logarithmic* with the increasing surface coverage^92^. The Temkin isotherm was expressed in the following *nonlinear* and *linearized form*

19 $${q}_{m}=\frac{RT}{{b}_{T}}\,\mathrm{ln}({K}_{T}{C}_{e})$$

20 $${q}_{m}=\left(\frac{RT}{{b}_{T}}\right)\mathrm{ln}\,{C}_{e}+\left(\frac{RT}{{b}_{T}}\right)\mathrm{ln}\,{K}_{F}$$

where *R* (8.314 J·mol^−1^·K^−1^) is the gas constant, *T* (K) is the absolute temperature, *K*_*T*_ (L·g^−1^) is the Temkin isotherm equilibrium binding constant, and *b*_*T*_ is the Temkin isotherm constant related to the heat of sorption (J·mol^−1^).

Dubinin-Radushkevich (D-R) model is a semi-empirical equation in which adsorption follows a pore filling mechanism with Gaussian energy distribution onto heterogeneous surfaces^93^. It has been successfully applied to describe the adsorption of gases on microporous adsorbents quantitatively^94^. The D-R isotherm was expressed in the following *nonlinear* and *linearized form*

21 $${q}_{e}={q}_{s}\exp (\,-\,{K}_{DR}{\varepsilon }^{2}),\,\varepsilon =RT\,\mathrm{ln}\left(1+\frac{1}{{C}_{e}}\right)$$

22 $$\mathrm{ln}\,{q}_{e}=\,\mathrm{ln}\,{q}_{s}-({K}_{DR}{\varepsilon }^{2}),\,\varepsilon =RT\,\mathrm{ln}\left(1+\frac{1}{{C}_{e}}\right)$$

where *K*_*DR*_ (mol^2^·kJ^−2^) and *q*_*s*_ (mg·g^−1^) is the D-R isotherm constant and the theoretical D-R isotherm saturation capacity, respectively.

**Figure 10:**
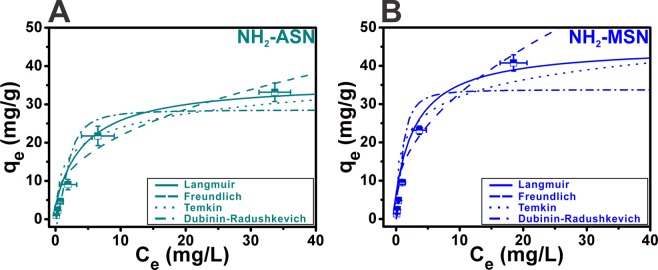
Cr(VI) adsorption isotherms of (**A**) NH_2_–ASNs and (**B**) NH_2_–MSNs at 25 °C. Best fits to data were successfully obtained using non-linear fits of the Langmuir, Freundlich, Temkin, and Dubinin-Radushkevich (D-R) models.

Both the *nonlinear* and *linearized form* of the four models were used to obtain best fits to adsorption equilibrium data of NH_2_–ASNs and NH_2_–MSNs at 25–55 °C and pH of 2.0. Figure 10 illustrates the results of best fits to the adsorption equilibrium data of NH_2_–ASNs and NH_2_–MSNs using the *nonlinear form* of the four models. Figure S6 shows the results of best fits to the adsorption equilibrium data of NH_2_–ASNs and NH_2_–MSNs using the *linearized form* of the four models. Table S3 summarizes the isotherm parameters and correlation coefficients such as *R*^2^ and *χ*^2^ obtained from best fits to data using the *linearized* and *nonlinear form* of the four models, respectively. The value of sum-of-squares (i.e., a measure of scatter of data points around the fitted curve) is used to compute *R*^2^ and *χ*^*2*^ is similarly calculated by minimizing the sum-of-squares of the nonlinear regression. The validity of each model was confirmed using *R*^*2*^ and *χ*^*2*^. According to *R*^*2*^ (0.95 < *R*^*2*^ < 0.99), the Langmuir and Freundlich models could fit the adsorption process reasonably well. However, applying the Langmuir model to the data of NH_2_–ASNs and NH_2_–MSNs resulted in improved fits with much smaller *χ*^*2*^ values than the Freundlich model. Consequently, our results suggest that the adsorption process of NH_2_–ASNs and NH_2_–MSNs follows the Langmuir isotherm better than the Freundlich model. A dimensionless separation factor *R*_*L*_, one of the characteristic parameters of the Langmuir isotherm, was defined as

23 $${R}_{L}=\frac{1}{1+{K}_{L}{C}_{0}}$$

where the value of *K*_*L*_ is the fitting parameters listed in Table S3 and the *C*_0_ is the initial Cr(VI) ion concentration (0 ≤ *C*_0_ ≤ 100). The value of *R*_*L*_ indicates the shape of the Langmuir isotherm to be either unfavorable (*R*_*L*_ > 1), linear (*R*_*L*_ = 1), favorable (0 < *R*_*L*_ < 1), or irreversible (*R*_*L*_ = 0)^95^. Figure S7 shows the *R*_*L*_ of NH_2_–ASNs and NH_2_–MSNs was less than 1. Note that the values of *R*_*L*_ from the Langmuir model and *1/n* from the Freundlich (Table S3) all lie between 0 and 1, which corroborates that the adsorption of Cr(VI) on both NH_2_–ASNs and NH_2_–MSNs is a favorable process with the maximum adsorption capacity of NH_2_–MSNs ~1.3 times greater than that of NH_2_–ASNs.

### Adsorption thermodynamics of NH_2_–ASNs and NH_2_–MSNs

Thermodynamic parameters of the adsorption process, such as change in standard free energy (*ΔG*°), enthalpy (*ΔH*°), and entropy (*ΔS*°) can be estimated from the data of the adsorption isotherm measured at various temperatures according to Eqs. 24, 25, and 26

24 $${K}_{0}=\mathop{\mathrm{lim}}\limits_{{C}_{e}\to 0}\frac{{q}_{e}}{{C}_{e}}$$

25 $$\varDelta {G}^{\circ }=-\,RT\,\mathrm{ln}\,{K}_{0}=\varDelta {H}^{\circ }-T\varDelta {S}^{\circ }$$

26 $$\mathrm{ln}\,{K}_{0}=-\,\frac{\varDelta {H}^{\circ }}{R}\frac{1}{T}+\frac{\varDelta {S}^{\circ }}{R}$$

where *K*_0_ is the adsorption distribution coefficient, which was obtained by plotting *ln(q*_*e*_*/C*_*e*_) versus *C*_*e*_ at different temperatures and extrapolating to zero *C*_*e*_^96^, *ΔH*° (kJ·mol^−1^) is the standard enthalpy change, *ΔS*° (J·mol^−1^·K^−1^) is the standard entropy change, *ΔG*° (kJ·mol^−1^) is the standard free energy change, *R* (8.314 J·mol^−1^·K^−1^) is the gas constant, and *T* (K) is the absolute temperature, respectively. *ΔH°* and *ΔS*° were obtained from the slope and intercept of the curve based on the well-known van’t Hoff equation (Eq. 27)

27 $$\mathrm{ln}\left(\frac{{K}_{0}({T}_{2})}{{K}_{0}({T}_{1})}\right)=-\,\frac{\varDelta {H}^{\circ }}{R}\left[\frac{1}{{T}_{2}}-\frac{1}{{T}_{1}}\right]$$

**Figure 11:**
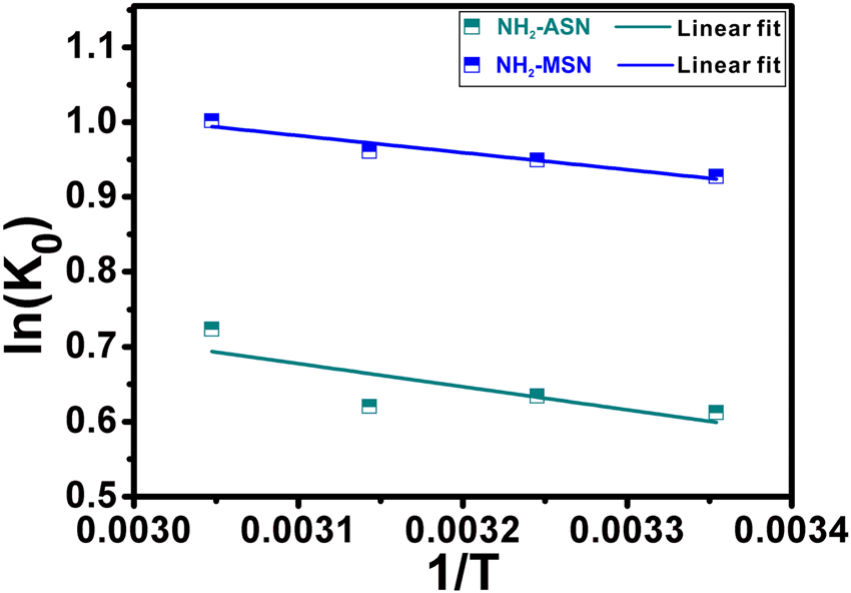
Plot of ln(*K*_0_) of NH_2_–ASNs (dark cyan) and NH_2_–MSNs (blue) versus inverse temperature (1/*T*). Best fits to data were successfully obtained using the linear regression of Eq. 26.

Figure 11 and Table 4 show the plot of *lnK*_*0*_ versus *1/T* and the corresponding thermodynamic parameters of Cr(VI) adsorption on NH_2_–ASNs and NH_2_–MSNs, repectively. According to Karthik *et al*., the adsorption process is mainly governed by electrostatic interaction between adsorption sites and adsorbing ions if the magnitude of *ΔH*° lies between 2.1 and 20.9 kJ·mol^−1^^97^. Note that *ΔH*° of NH_2_–ASNs and NH_2_–MSNs listed in Table 4 was 2.6 and 1.9 kJ·mol^−1^, respectively. These values were nearby the range (2.1 kJ·mol^−1^ ≤ *ΔH*° ≤ 20.9 kJ·mol^−1^), indicating the process was likely electrostatic physisorption. The positive values of *ΔH*° revealed the endothermic nature of the adsorption process, which was consistent with the results of temperature-dependent adsorption isotherms. In general, all *ΔG*° values of NH_2_–ASNs and NH_2_–MSNs were negative for the temperatures from 25 to 55 °C, exhibiting that the adsorption was a spontaneous process. The *ΔG*° of NH_2_–MSNs was approximately ~1.5 times greater than that of NH_2_–ASNs likely due to the presence of nanopores. The positive value of *ΔS*° suggests the increased randomness at the liquid-solid interface during Cr(VI) adsorption likely indicates the process is an entropy-driven process rather than enthalpy^97^.

**Table 4:** Thermodynamic parameters for Cr(VI) adsorption on NH_2_–ASNs and NH_2_–MSNs obtained from the linear regression shown in Fig. 11.

Temperature (K)	NH_2_-ASN			NH_2_-MSN		
	ΔG (kJ mol^−1^)	ΔH (kJ mol^−1^)	ΔS (J mol^−1^ K^−1^)	ΔG (kJ mol^−1^)	ΔH (kJ mol^−1^)	ΔS (J mol^−1^ K^−1^)
298.15	−1.5792	2.5654	13.587	−2.2985	1.8994	14.052
308.15	−1.6251			−2.4317		
318.15	−1.6409			−2.5412		
328.15	−1.9742			−2.7329		

### Effect of foreign ions on Cr(VI) removal efficiency of NH_2_–ASNs and NH_2_–MSNs

**Figure 12:**
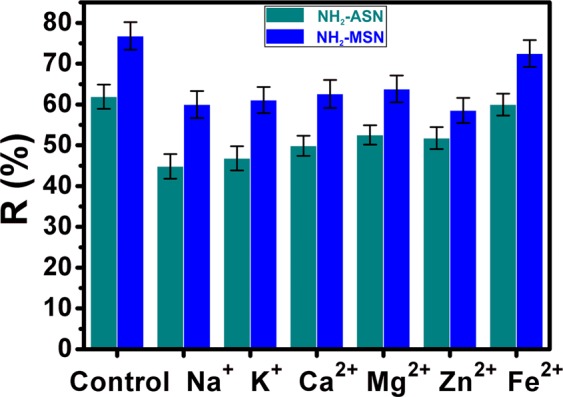
Effect of foreign ions on Cr(VI) removal by NH_2_–ASNs (dark cyan) and NH_2_–MSNs (blue).

Figure 12 shows the effect of foreign ions such as Na^+^, K^+^, Ca^2+^, Mg^2+^, Zn^2+^, and Fe^2+^ on the Cr(VI) removal efficiency (*R*) of NH_2_–ASNs and NH_2_–MSNs with Cr(VI) concentration of 100 mg/L at 25 °C. The results indicated that the adsorption capacity of NH_2_–ASNs and NH_2_–MSNs slightly declined with the presence of the foreign ions by no more than 28 and 22% of the control, respectively. Note that monovalent cations exerted a slightly more influence on the Cr(VI) removal performance of NH_2_–ASNs and NH_2_–MSNs than multivalent ions. The adverse effect of foreign cations on the uptake of Cr(VI) ions has been attributed to the presence of ion exchange during the adsorption process^98,99^. Under the circumstance of increased number of the foreign cations, the electrostatic attraction between the negatively charged Cr(VI) ions and the positively charged NH_3_^+^–groups becomes weaker, resulting in inhibiting the adsorption of Cr(VI) ions, and thus the decline of *R*. Nevertheless, our findings suggest that NH_2_–MSNs exhibit a robust and high enough Cr(VI) removal performance marginally better than NH_2_–ASNs under the influence of foreign ions.

### Desorption-regeneration of NH_2_–ASNs and NH_2_–MSNs

**Figure 13:**
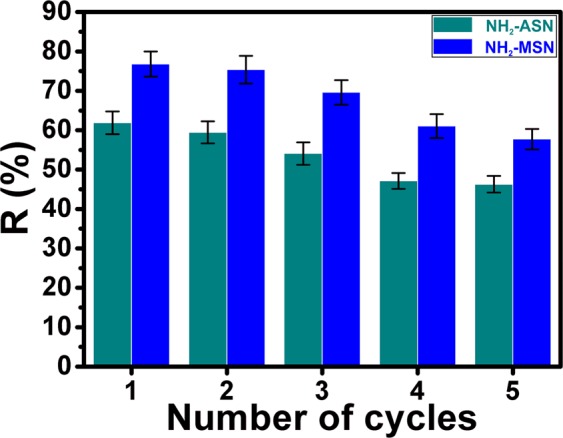
Desorption-regeneration of NH_2_–ASNs (dark cyan) and NH_2_–MSNs (blue) for Cr(VI) removal.

Desorption-regeneration experiments of NH_2_–ASNs and NH_2_–MSNs were carried out to assess the feasibility of their recovery and future usage. Figure 13 shows the removal efficiencies (*R*) of NH_2_–ASNs and NH_2_–MSNs after five cycles of desorption-regeneration with 0.1 M HCl solution at temperature and pH of 25 °C and 2.0. The removal efficiency (*R*) of NH_2_–ASNs and NH_2_–MSNs after five regenerations had slightly decreased from 61.9 to 46.3 and 76.8 to 57.8%, respectively. The reduction of their removal efficiency after five cycles was only less than 25% of the starting value, which suggests that NH_2_–ASNs and NH_2_–MSNs may have a promising recycling property for Cr(VI) removal.

## Conclusions

We present a comprehensive analysis of Cr(VI) adsorption and removal on both bare and NH_2_-functionalized ASNs and MSNs in aqueous environments. Highly monodisperse ASNs and MSNs of approximately equal sizes were successfully synthesized using modifications of the Stöber method and subsequently surface functionalized with amine groups. The specific surface area of MSNs (as obtained by N_2_ sorption measurements) was twice as large as that of ASNs, and the NH_2_–functional group loading value of APTES reacted MSNs was four times greater than that of APTES reacted ASNs. Further analyses of N_2_ sorption measurements using a simple geometric scaling approach revealed that more than 70% of the total specific surface area of MSNs and NH_2_–MSNs was attributable to nanopores within MSNs and NH_2_–MSNs, respectively. Furthermore, the specific surface areas of ASNs and MSNs decreased as APTES surface loading increased. Cr(VI) adsorption and removal efficiencies were determined using a series of kinetic adsorption batch experiments. The kinetics of the Cr(VI) adsorption processes exhibited a rapid adsorption whereby ~90% of Cr(VI) adsorption was achieved within a minute. Fits with experimental data of the pseudo-first and pseudo-second order kinetic models were investigated, and the intraparticle diffusion model was also applied in an attempt to understand the mechanism of the adsorption process. Best fits with experimental data were obtained by applying nonlinear fits to the pseudo-second order model. The outcomes of fitting processes were verified by evaluating standard residuals (SRs) rather than correlation coefficients (*R*^2^). Application of the intraparticle diffusion model also resulted in good fits with kinetic data and indicated adsorption might involve multiple steps. The adsorption isotherms were analyzed as a function of temperature by nonlinear and linearized regression, and the Langmuir model resulted in best fits to the isotherms. The thermodynamic parameters indicated that the adsorption of Cr(VI) on NH_2_–ASNs and NH_2_–MSNs was endothermic in nature and an entropy-driven spontaneous process. The results of desorption-regeneration experiments suggest that NH_2_–ASNs and NH_2_–MSNs may have a promising potential for recycling. Although the specific surface areas of NH_2_–ASNs and NH_2_–MSNs were lower than those of ASNs and MSNs, NH_2_–ASNs and NH_2_–MSNs had significantly higher Cr(VI) adsorption capacities (34.0 and 42.2 mg·g^−1^, respectively) and removal efficiencies (61.9 and 76.8%, respectively). These values compare reasonably well with those reported for powder-like mesoporous silica, Stöber silica, and hybrid silica nanoparticles^27,39,76,100^. Furthermore, these results suggest both the introduction of nanoporous architecture and appropriate chemical functionalization of nanoparticle surfaces are essential for enhancing Cr(VI) adsorption and removal process.

The findings of the current study are significant in two respects. First, they show silica nanoparticles possessing well-defined sizes and shapes with narrow distributions can be produced and chemically functionalized^34^. The spherical geometry of the nanoparticles provided admittedly simplified modeling interactions between them and Cr(VI) and facilitated the study of Cr(VI) adsorption processes. In the present study, we used model systems and material characterization tools to investigate Cr adsorption on silica nanoparticles and the results suggest a structural and chemical rationale for enhanced Cr(VI) adsorption and removal. Second, the NH_2_–ASNs and NH_2_–MSNs examined converted most of the adsorbed Cr(VI) to relatively non-toxic Cr(III) at levels comparable to or marginally better than those reported for similar mesoporous silica systems^6,38,75,76,100^. Our results suggest Cr(VI) reduction is caused by a mixed adsorption-partial reduction process that may be characteristic of functionalized nanoparticle-based systems^6,73,101,102^. Understanding the detailed mechanism of Cr(VI) to Cr(III) conversion represents a meaningful step toward the goal of removing toxic Cr(VI) from wastewater environments.

## Data Availability

All data generated or analyzed during this study are included within the article and are available from the corresponding author upon request.

## Supporting Information

Supporting Information.
