# Supplementary material for: A systematic study of hexavalent chromium adsorption and removal from aqueous environments using chemically functionalized amorphous and mesoporous silica nanoparticles
Source: Sci Rep. 2020 Mar 27;10:5558. doi: 10.1038/s41598-020-61505-1 (PMC7101345; doi:10.1038/s41598-020-61505-1)
Supplement: Supplementary file 1 — Supporting Information. [file 41598_2020_61505_MOESM1_ESM.docx]

**Supplementary information for**

**A systematic study of hexavalent chromium adsorption and removal from aqueous environments using chemically functionalized amorphous and mesoporous silica nanoparticles**

Eun-Hye Jang^†§^, Seung Pil Pack^‡^, Il Kim^§^, and Sungwook Chung^†*^

**^†^**School of Chemical and Biomolecular Engineering, Pusan National University,

2 Busandaehak-ro 63beon-gil, Geumjeong-gu, Busan, 46241, South Korea

^‡^Department of Biotechnology and Bioinformatics, Korea University, 2511 Sejong-Ro, Sejong, 30019, South Korea

^§^Department of Polymer Science and Engineering, Pusan National University, 2 Busandaehak-ro 63beon-gil, Geumjeong-gu, Busan, 46241, South Korea

*Author to whom correspondence should be addressed.

E-mail: [sungwook.chung@pusan.ac.kr](mailto:sungwook.chung@pusan.ac.kr)

**
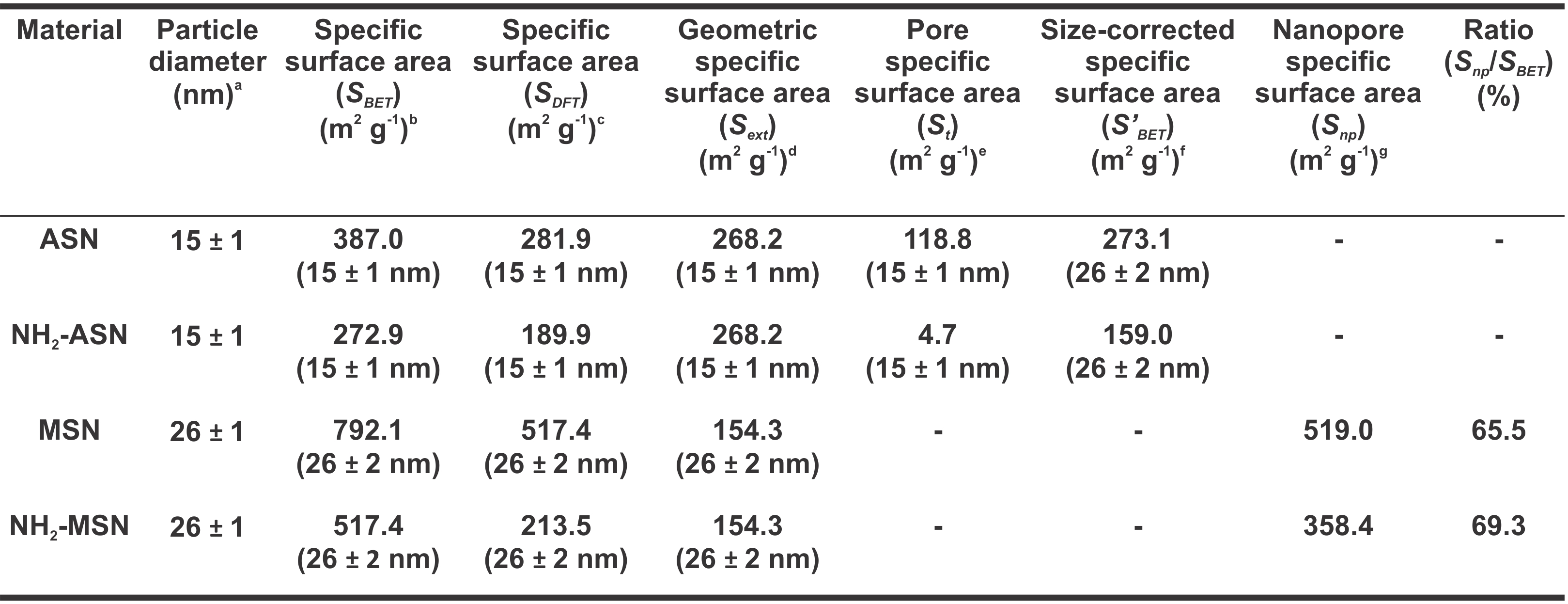
**

**Table S1.** Specific surface areas (SSAs) determined using the BET model (*S_BET_*), calculated specific surface areas by the DFT method (*S_DFT_*), calculated geometric specific surface area (*S_ext_*) based on the sizes of ASNs, NH_2_–ASNs, MSNs, and NH_2_–MSNs, calculated pore specific surface areas (*S_t_*), calculated size-corrected specific surface areas (*S′_BET_*), calculated nanopore specific surfaces areas (*S_np_*), and *S_np_* to *S_BET_* ratios of ASNs, NH_2_–ASNs, MSNs, and NH_2_–MSNs.


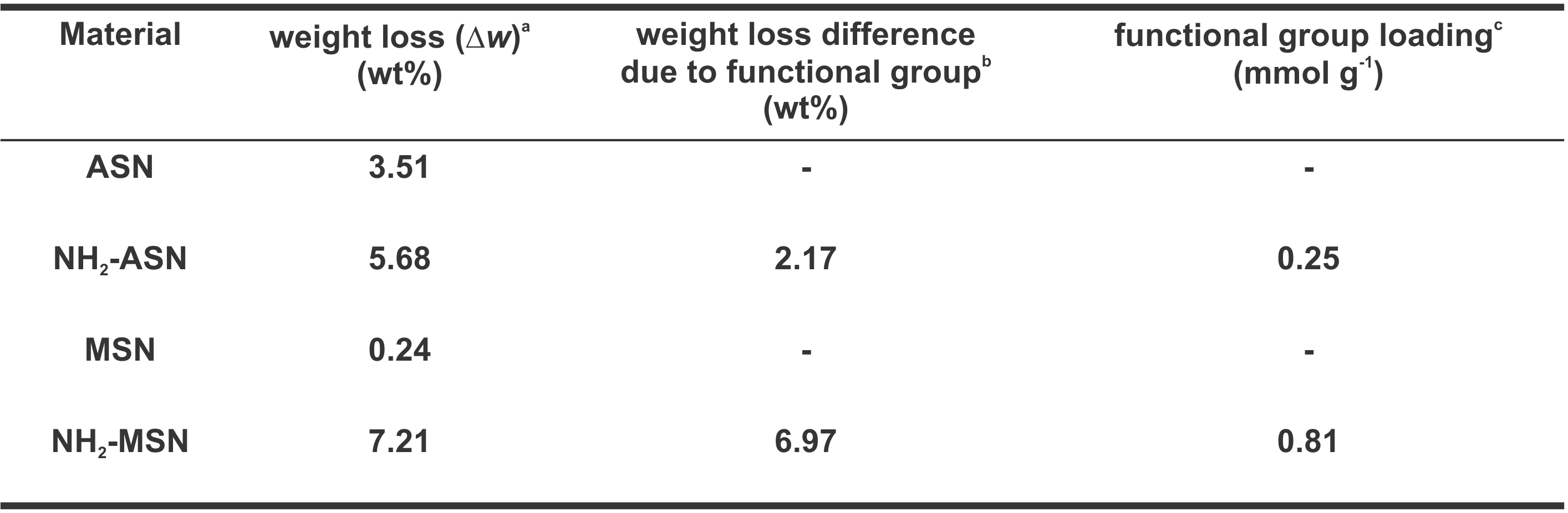


**Table S2.** Weight losses and functional group loadings of ASNs, NH_2_–ASNs, MSNs, and NH_2_–MSNs as determined by thermal gravimetric analysis/differential thermal analysis (TGA/DTA) (curves are shown in Figure S2).


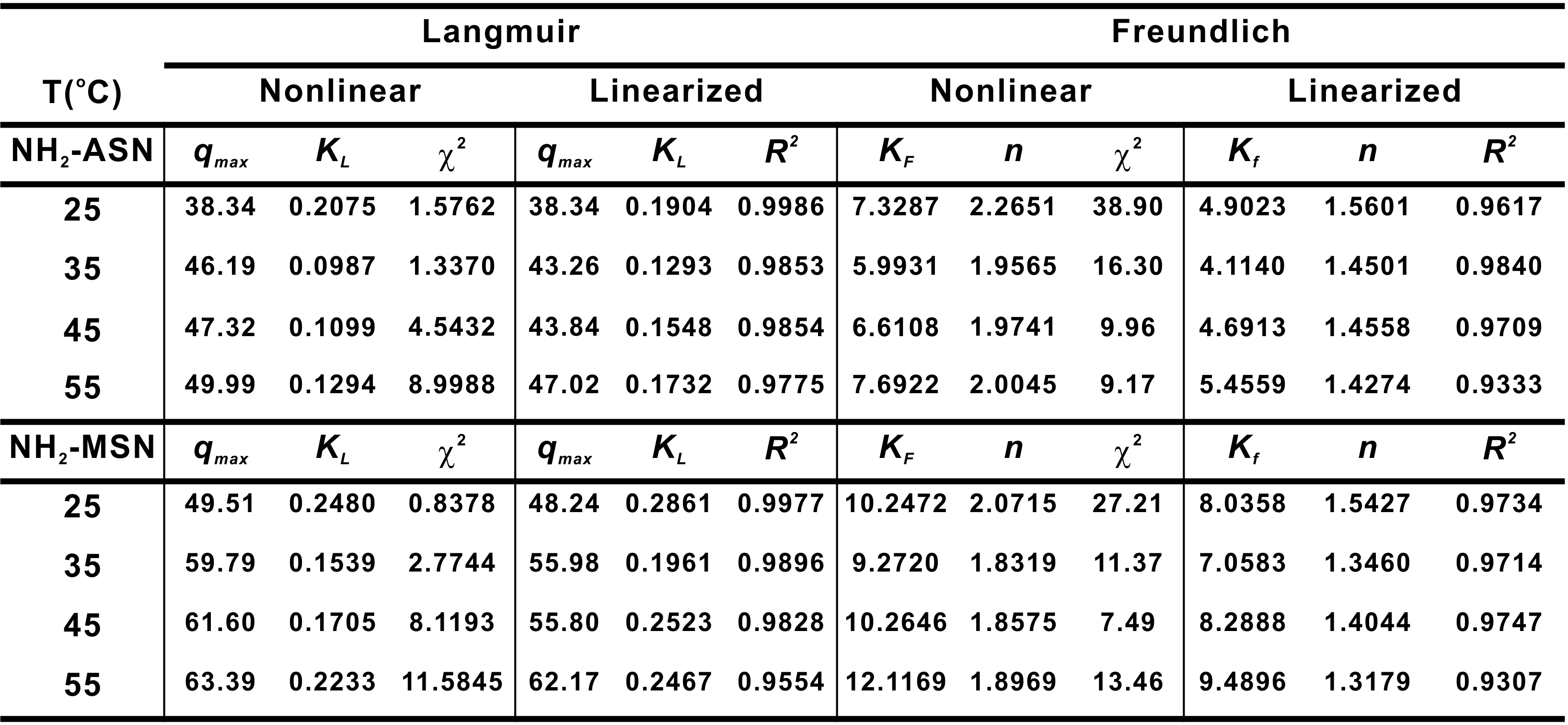


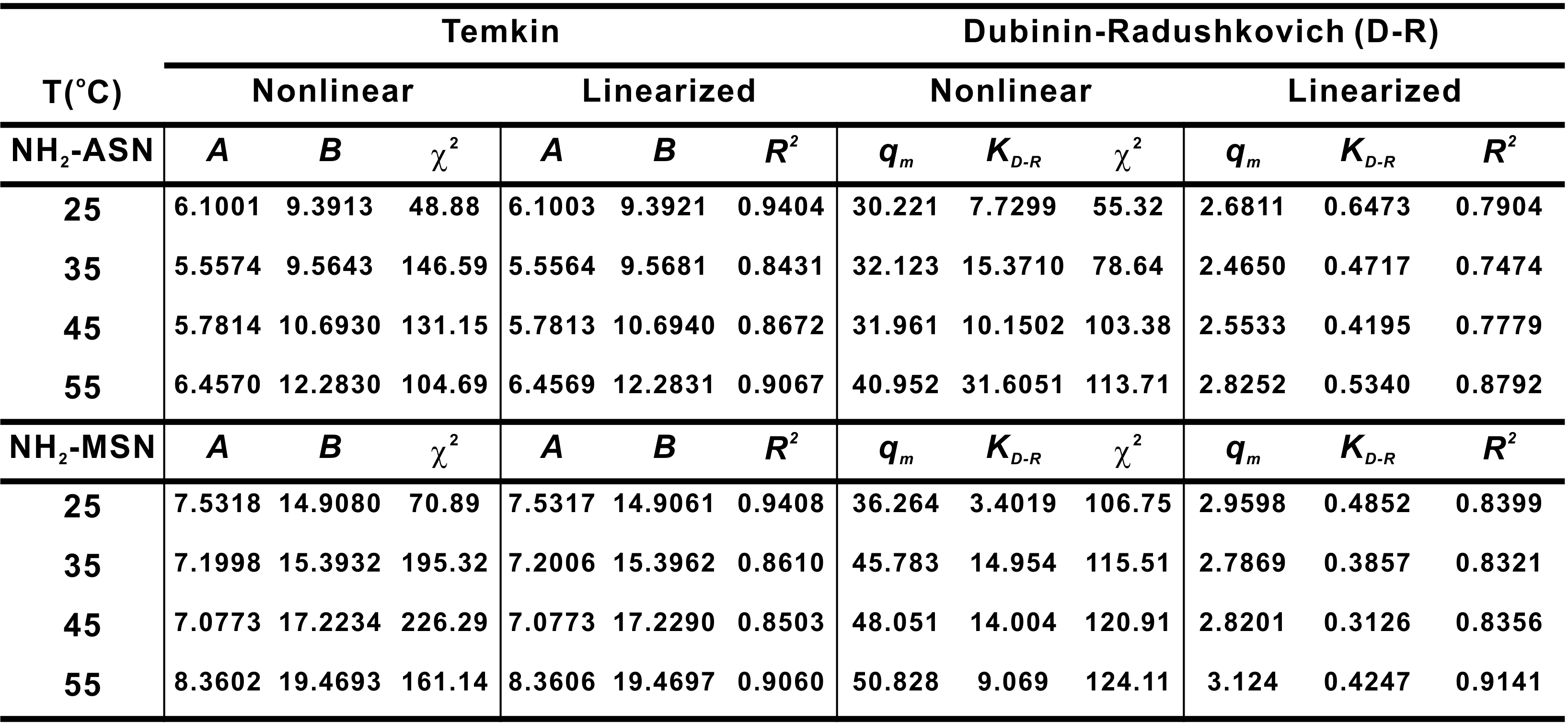


**Table S3.** Isotherm parameters for the adsorption of Cr(VI) on NH_2_–ASNs and NH_2_–MSNs at various temperatures obtained from best fits to data using the nonlinear and linearized form of the Langmuir, Freundlich, Temkin, and Dubinin-Radushkevich (D-R) models.


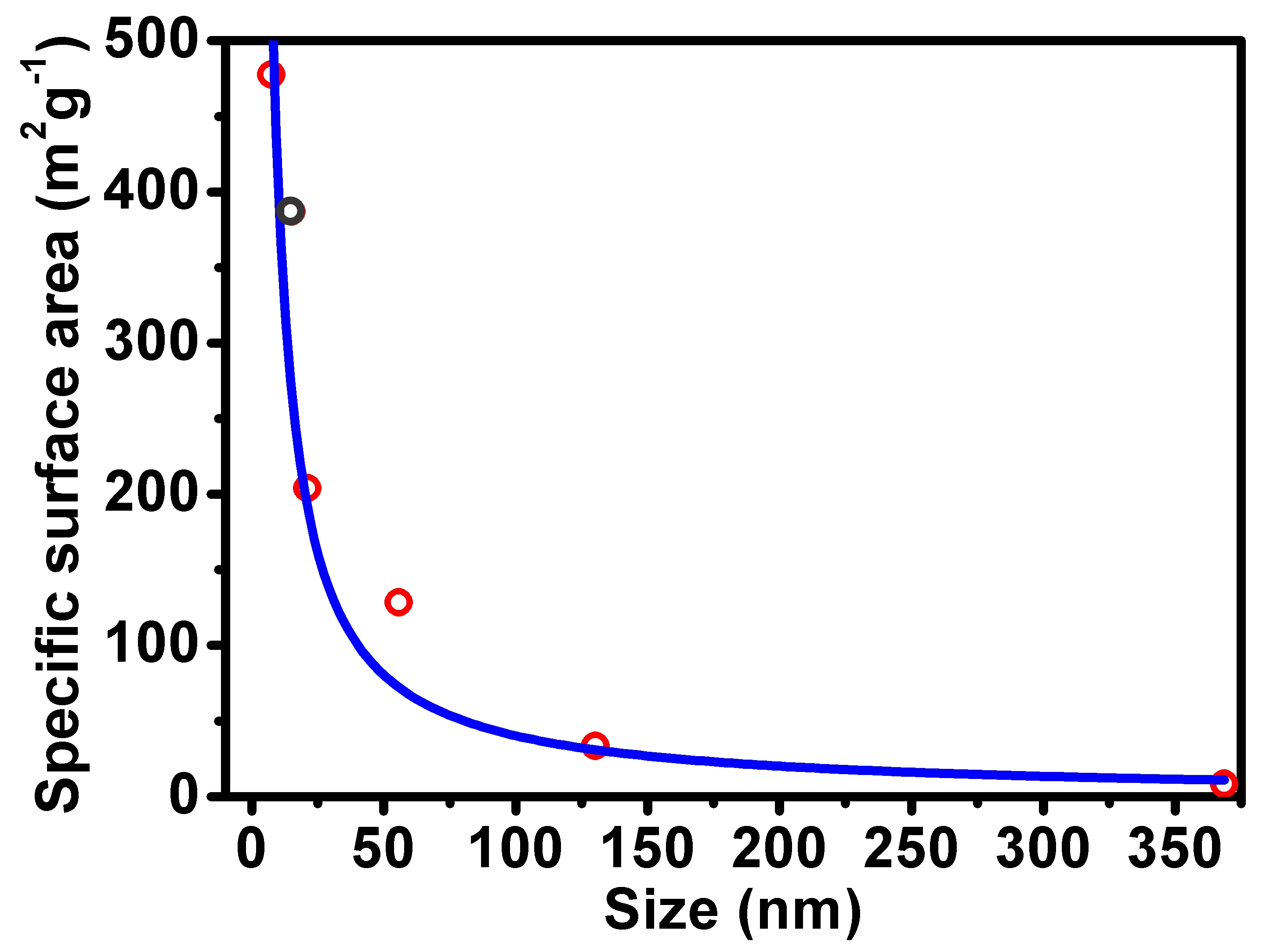


**Figure S1.** Plot of measured specific surface areas (SSAs) versus ASN particle sizes.


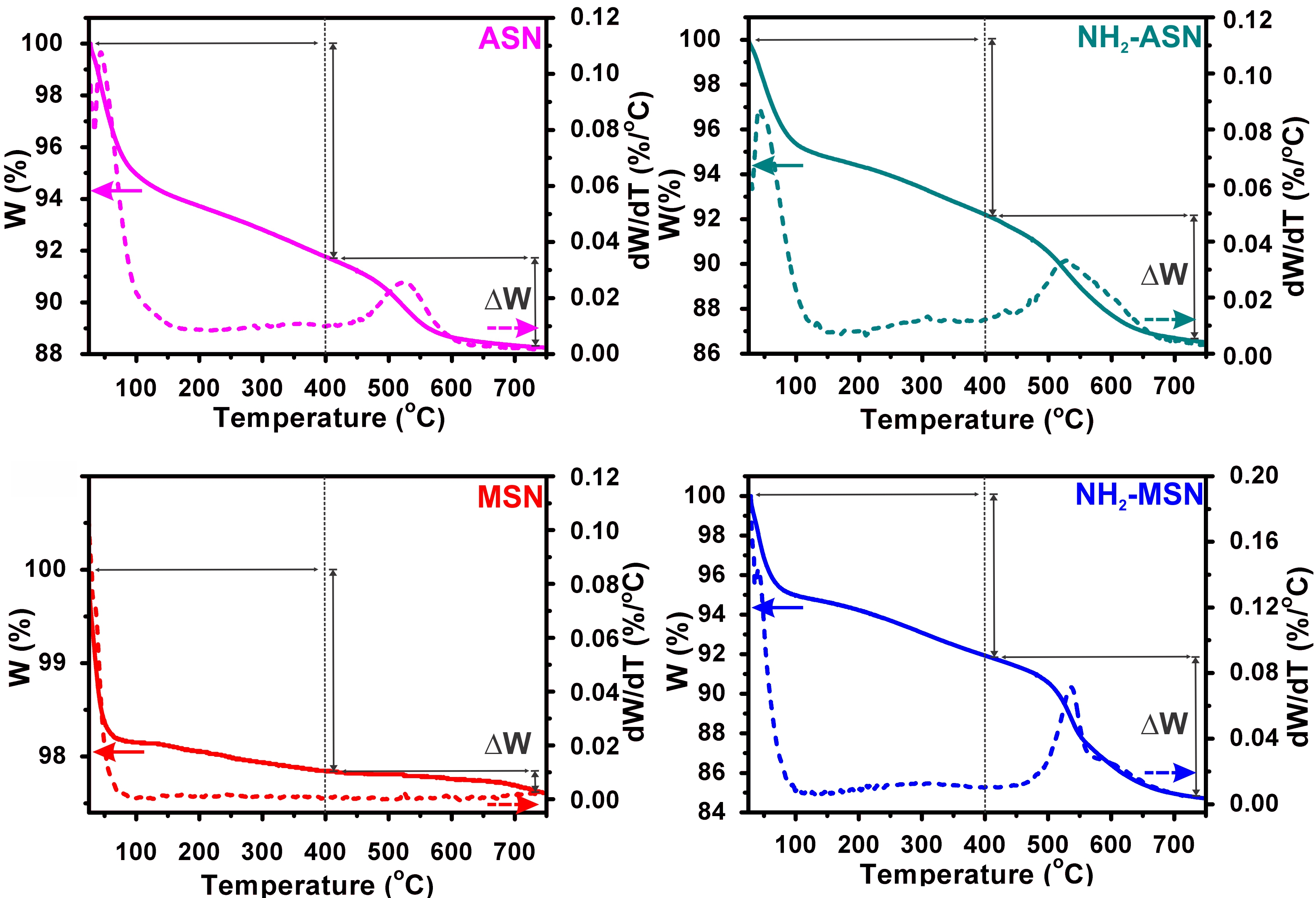


**Figure S2.** TGA/DTA curves of ASNs (magenta), NH_2_–ASNs (dark cyan), MSNs (red), and NH_2_–MSNs (blue) showing weight losses as a function of temperature.


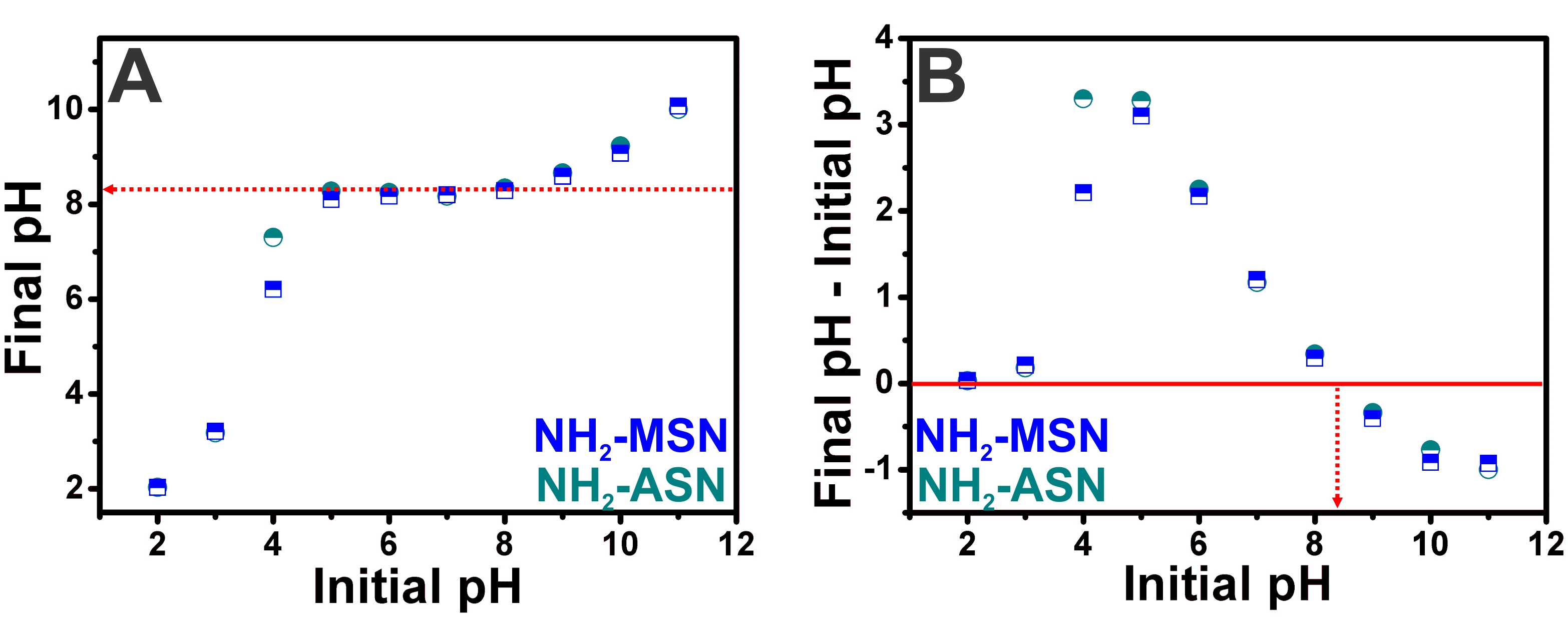


**Figure S3.** pH at the point of zero charge (pH_pzc_) of NH_2_–ASNs and NH_2_–MSNs.


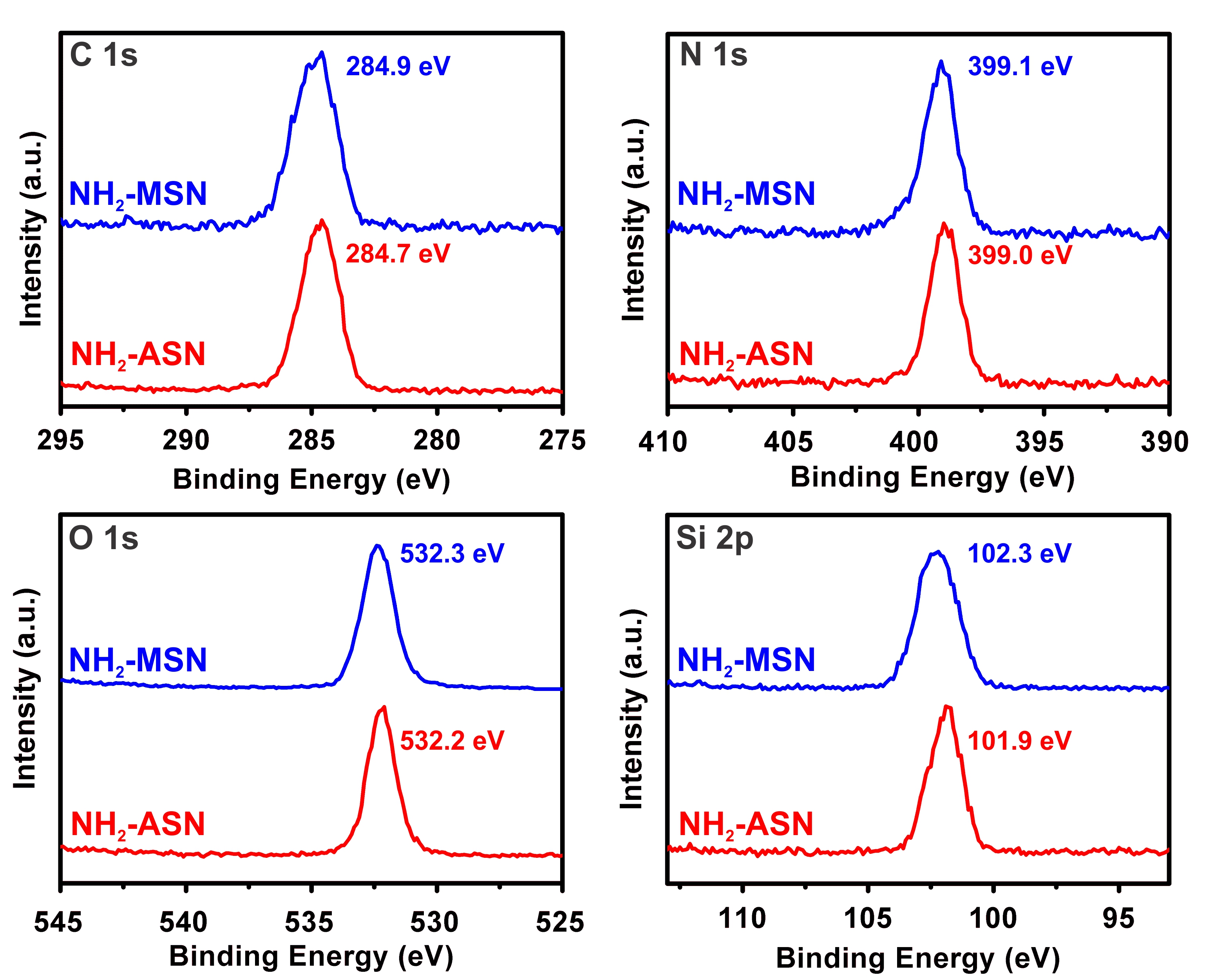


**Figure S4.** High resolution XPS spectra of the C 1s, N 1s, O 1s, and Si 2p peaks of NH_2_–ASNs and NH_2_–MSNs.


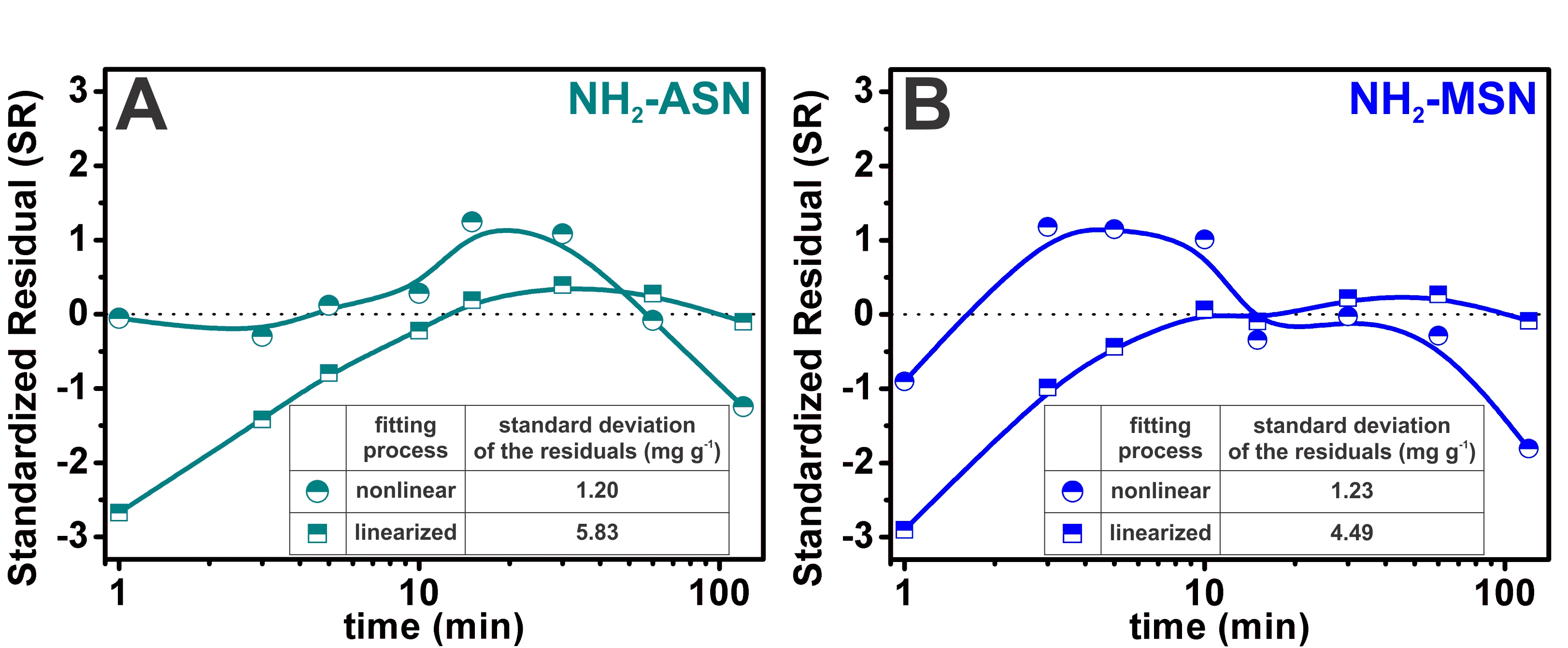


**Figure S5.** Plots of standardized residuals (SRs) of the experimental values of *q_e_* for Cr(VI) adsorption by (A) NH_2_–ASNs (dark cyan) and (B) NH_2_–MSNs (blue) versus time.


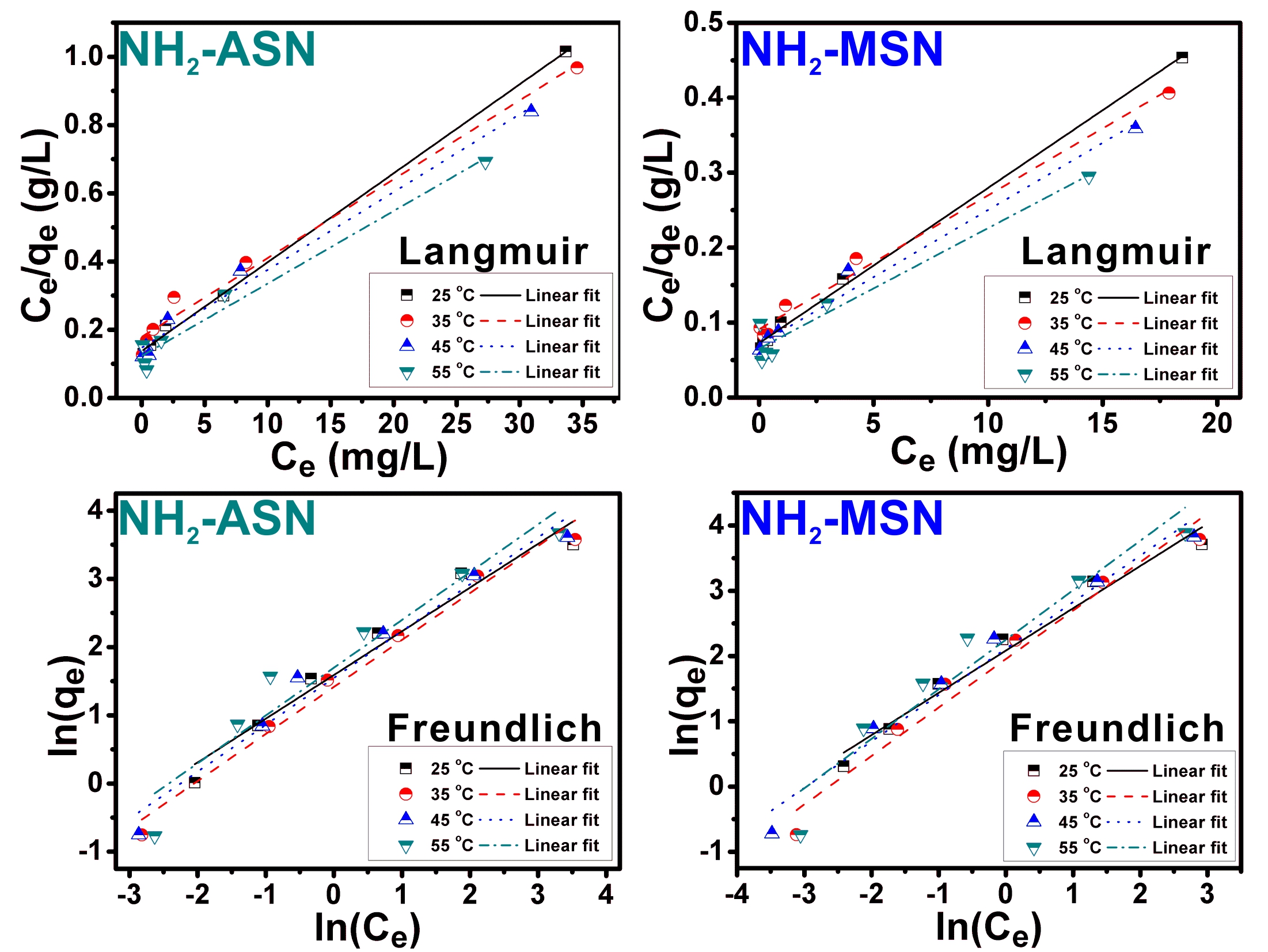


**Figure S6.** Cr(VI) adsorption isotherms of NH_2_–ASNs and NH_2_–MSNs at 25, 35, 45, and 55 °C. Best fits to data were successfully obtained using linearized fits of the Langmuir, Freundlich, Temkin, and Dubinin-Radushkevich (D-R) models.


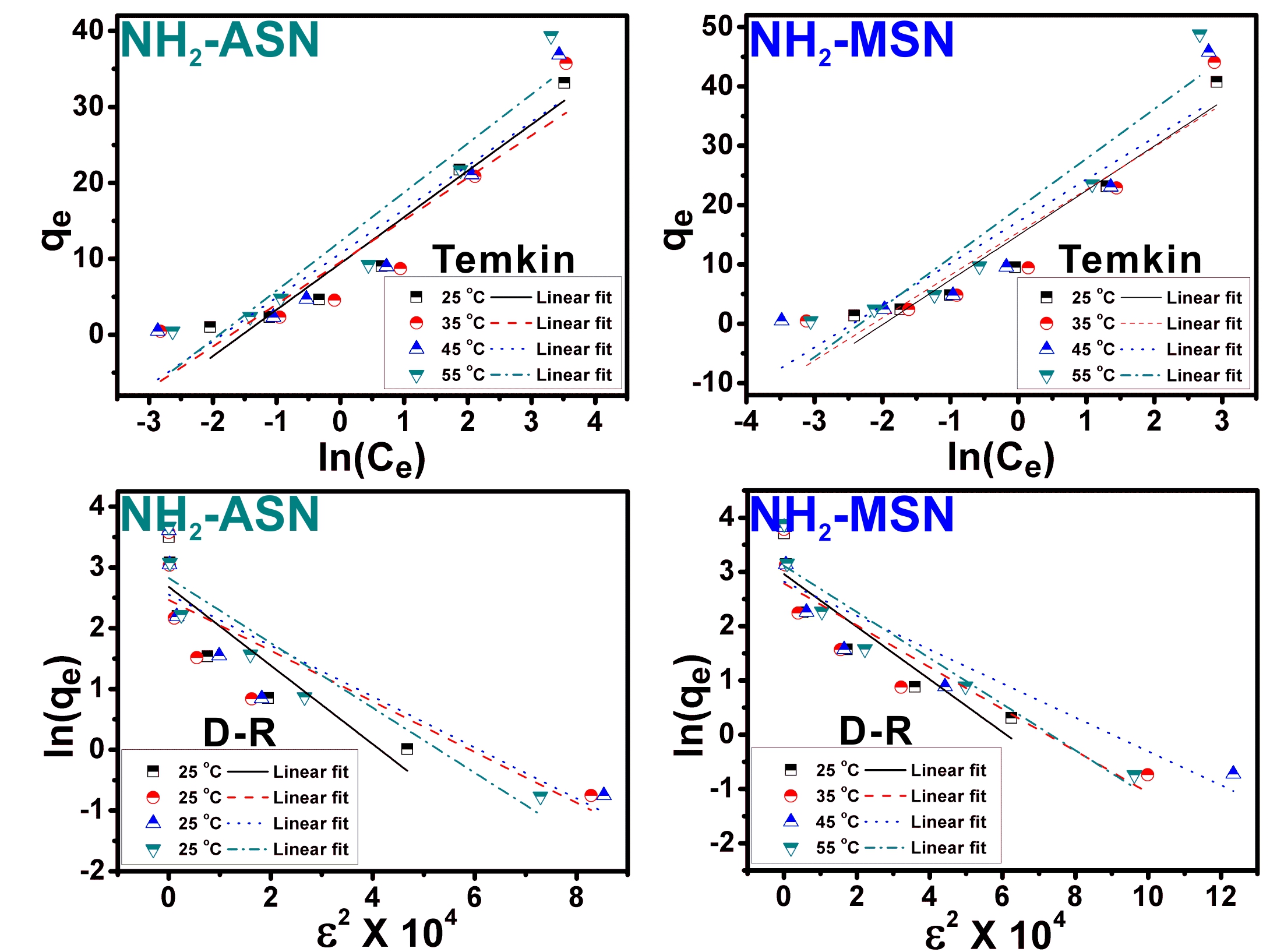


**Figure S6.** (cont.) Cr(VI) adsorption isotherms of NH_2_–ASNs and NH_2_–MSNs at 25, 35, 45, and 55 °C. Best fits to data were successfully obtained using linearized fits of the Langmuir, Freundlich, Temkin, and Dubinin-Radushkevich (D-R) models.


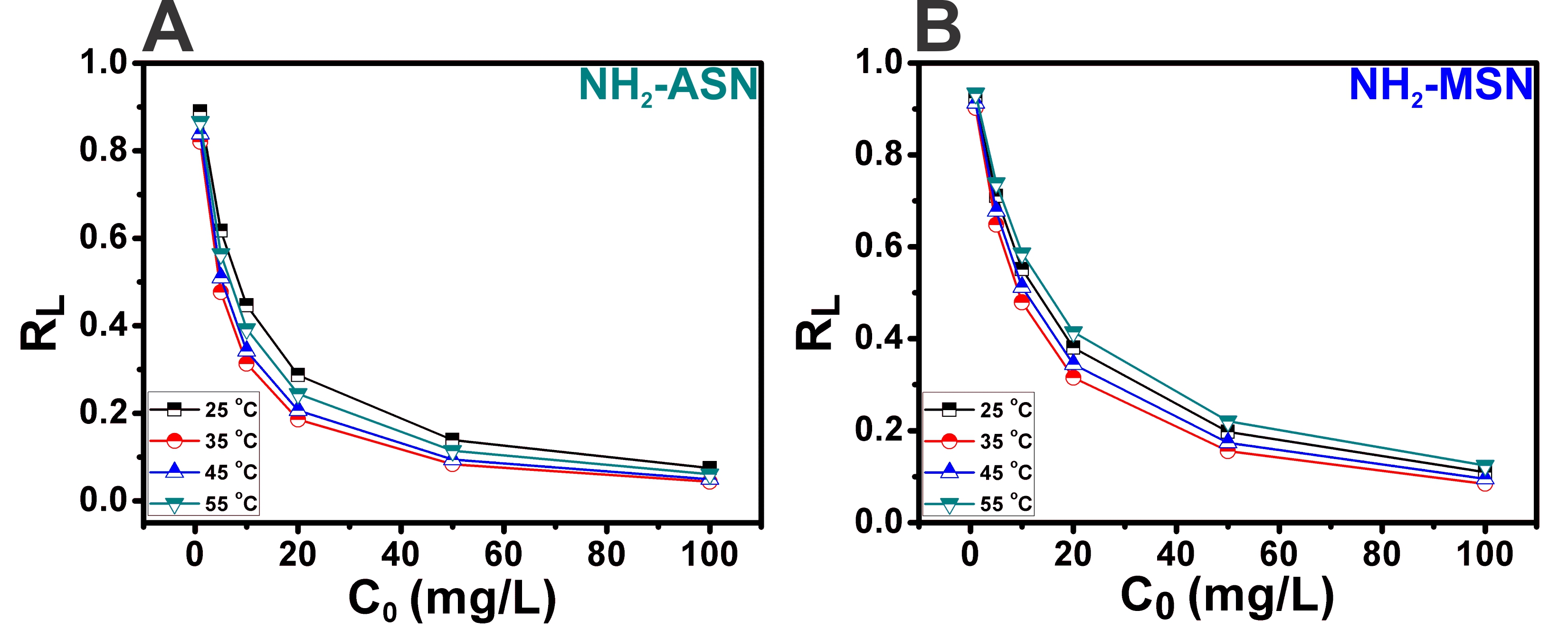


**Figure S7.** Plots of *R_L_* of (A) NH_2_–ASNs and (B) NH_2_–MSNs versus initial Cr(VI) concentrations at various temperatures.
